# Discovery of human cell selective effector molecules using single cell multiplexed activity metabolomics

**DOI:** 10.1038/s41467-017-02470-8

**Published:** 2018-01-02

**Authors:** David C. Earl, P. Brent Ferrell, Nalin Leelatian, Jordan T. Froese, Benjamin J. Reisman, Jonathan M. Irish, Brian O. Bachmann

**Affiliations:** 1 Department of Chemistry, Vanderbilt University, 7330 Stevenson Center, Station B 351822, Nashville, TN 37235 USA; 2 Department of Medicine, Vanderbilt University Medical Center, 1161 21st Avenue South, D-3100 Medical Center North, Nashville, TN 37232 USA; 3 Department of Cell and Developmental Biology, Vanderbilt University, 465 21st Avenue South, Nashville, TN 37232 USA; 4 Vanderbilt-Ingram Cancer Center, Vanderbilt University Medical Center, 2220 Pierce Avenue, Nashville, TN 37232 USA; 5 Department of Pathology, Microbiology and Immunology, Vanderbilt University Medical Center, 1161 21st Avenue South, D-2220 Medical Center North, Nashville, TN 37232 USA

## Abstract

Discovering bioactive metabolites within a metabolome is challenging because there is generally little foreknowledge of metabolite molecular and cell-targeting activities. Here, single-cell response profiles and primary human tissue comprise a response platform used to discover novel microbial metabolites with cell-type-selective effector properties in untargeted metabolomic inventories. Metabolites display diverse effector mechanisms, including targeting protein synthesis, cell cycle status, DNA damage repair, necrosis, apoptosis, or phosphoprotein signaling. Arrayed metabolites are tested against acute myeloid leukemia patient bone marrow and molecules that specifically targeted blast cells or nonleukemic immune cell subsets within the same tissue biopsy are revealed. Cell-targeting polyketides are identified in extracts from biosynthetically prolific bacteria, including a previously unreported leukemia blast-targeting anthracycline and a polyene macrolactam that alternates between targeting blasts or nonmalignant cells by way of light-triggered photochemical isomerization. High-resolution cell profiling with mass cytometry confirms response mechanisms and is used to validate initial observations.

## Abstract (web summary)

Bioactive metabolites from plant and microbial extracts hold therapeutic potential. Here, the authors combine untargeted metabolomic arrays with flow cytometry-based single cell response profiling and identify metabolites with cell subset-specific activities in the bone marrow from an AML patient.

## Introduction

A metabolome is the sum of primary and secondary metabolites produced by an organism in its environment. Constitutive metabolites are capable of interacting intra- and extracellularly with receptors and active sites within DNA^1–3^, RNA^4,5^, and proteins^6,7^, and metabolites are therefore close partners in growth, homeostasis, and signaling in heterogeneous environments^8–12^. Chemical communication mediated via the inventory of an organism’s cellular metabolites therefore defines an important molecular axis of interaction within and between organisms^13^. Tapping into this communication system has become a central empirical activity in chemical biology and has repeatedly illuminated molecular solutions to problems with significant clinical relevance, such as the discovery of new bioeffector antibiotics and chemotherapeutics^14,15^. Tools to map novel bioeffector molecules to functional roles in responding cells—i.e., to identify bioeffector mechanism class—have been adapted into single-cell assays^16,17^ that stratify clinical outcomes and predict treatment responses^18–23^. Together with cellular barcoding^24,25^ and single-cell chemical biology assays^26^, the recent advances in cytomics have raised the exciting possibility of undertaking personalized metabolomic response profiling and bioeffector mechanism class identification in primary human tissue biopsies obtained for clinical research^16,27,28^.

Despite the centrality of metabolite functional analysis, the development of a generalizable omics-scale solution for uncovering the functional roles of secondary metabolites within disease-relevant cellular contexts remains a substantial challenge^29^. It is now possible to convert biological extracts (e.g., of microbial culture, plant/tissue origin) into highly characterized chromatographic microtiter arrays by split flow liquid chromatographic mass spectrometry^30^. The biological characterization of such untargeted metabolomic arrays results in the generation of “bioactivity chromatograms”, and correlation analysis to matched extracted ion current (EIC) mass chromatograms identifies candidate metabolites linked to measured bioassay targets. However, per-well single-assay modalities greatly limit the efficiency of this approach, and targeted biochemical assays or phenotypic assays against cell lines reveal only a fraction of significant roles of metabolites in arrays.

Signaling profiles of primary cancer cells measured using phospho-specific flow cytometry (phospho-flow) have been shown to stratify the outcome of acute myeloid leukemia (AML)^20,23^ and B cell non-Hodgkin’s lymphoma^18,19^ based on signaling network responses to environmental cues, such as cytokines. Single-cell chemical biology assays have also been developed for fluorescence cytometry^26^ and mass cytometry^31^ to characterize pathway and cell-type-specific responses to small molecules. Fluorescence cytometry has the advantage of cellular throughput and more robust barcoding potential^32^, whereas mass cytometry has the power to track more than 35 key markers of AML cell phenotype and function simultaneously^23,33,34^. These assays rely on cellular barcoding to multiplex a large number of variables representing stimulation conditions, compounds, dosages, or timepoints^24,25,35^. Such cytomic approaches are further strengthened by recently developed computational tools to reveal and characterize changes in cell subsets^33,36,37^.

Here, a combination of (1) phospho-flow, (2) single-cell chemical biology, and (3) cellular barcoding was matched with (4) metabolomic arrays to identify natural product secondary metabolites that specifically target primary human leukemia cells and spare adjacent nonmalignant immune cells. This activity-metabolomics platform is termed multiplexed activity metabolomics (MAM) and comprises a system for single-cell metabolome-scale analysis of bioactivity using human cells from primary tissue biopsies in a high-throughput screening-compatible microtiter format. This untargeted assay modality samples a cross section of biological responses in a heterogeneous mixture of cells representing an in vivo human tissue environment and has the potential to identify disease-relevant bioactive metabolites within metabolomic arrays.

## Results

### Cytometry-enabled MAM platform

**Fig. 1:**
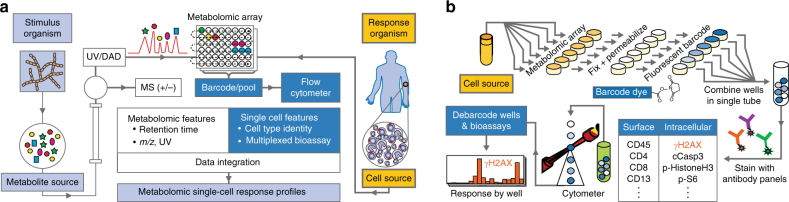
Metabolome functional analysis by multiplexed activity metabolomics (MAM). **a** High-data-content metabolomic arrays are generated in replicate from a “stimulus” organism via split-flow polarity switching chromatography mass spectrometry. A suspension of disaggregated tissue cells from a “response” organism (human) is added to the metabolomic array. **b** Flow cytometric cell barcoding and multiplexed immunoassays are used to identify multiple cell type/subtype-specific biological responses to metabolites in the array. Correlation analysis of the resulting bioactivity and UV/ESI/MS(±) data generate putative functional activities for metabolites. An example of MAM with a natural product producing actinomycete and a cell preparation derived from an AML patient including, but not limited to lymphocytes, monocytes, and leukemia blasts, as well as their subtypes is illustrated here

The MAM workflow (Fig. 1) first generates a metabolomic array in microtiter plate format via reversed-phase liquid chromatographic separation of a crude biological extract produced by a “stimulus” organism. A portion of the effluent is diverted to a polarity-switching electrospray mass spectrometric analyzer (ESI-MS) and the remainder of the effluent to a microtiter plate fraction collector after passing through a UV/Vis diode array detector. Following evaporation and resuspension of collected fractions, cell preparations from a “response” organism (e.g., humans, represented by tissue cells) are added to the microtiter wells for incubation with the metabolomic fractions to induce cellular responses (Fig. 1a). Cells within wells are then stained for viability, fixed, and permeabilized, and fluorescent cell barcoding (FCB, Fig. 1b) is used to label the well contents via differential staining of cells with *N*-hydroxysuccinimide (NHS) ester-functionalized fluorescent dyes^3^. Thus, “barcoded” cells in the microtiter wells are then pooled and stained with multiple fluorescent antibodies to quantitate cell status and targeted cell-type-specific responses to metabolites. Critically, flow cytometric gating based on the barcoding fluorophores facilitates the assignment of cells to their original coordinates on the microtiter plate metabolite array (i.e., “deconvolutes” treatment conditions for each cell), yielding simultaneous bioassay marker quantitation per well for each targeted antibody–fluorophore conjugate. Barcoding enables high-throughput antibody assays by using a fraction of antibody reagents compared to a microtiter format, and pooling also ensures the uniformity of antibody staining of cells across all wells, decreasing experimental variation. The result is a multiplexed series of well coordinate-linked immunoassay profiles running through the metabolomic fraction array. Metabolomic features (positive and negative *m/z*, UV, and retention time), which are generated via EIC analysis of all significant detected metabolites, are then manually or automatically correlated with bioactivity features, which are peaks generated from bioassay profiles across wells.

**Fig. 2:**
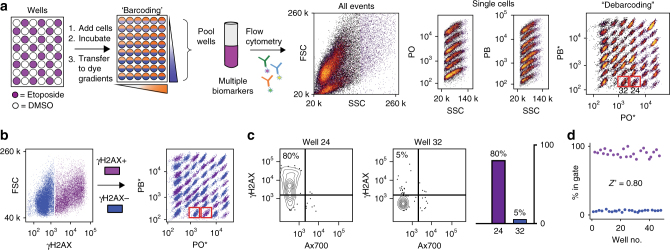
Validation of a 48-well single-cell chemical biology assay for DNA damage response mechanism class. Design of a checkerboard validation experiment using Kasumi cells and a DNA-active natural product. **a** Overview of 48-well fluorescent cell barcoding and debarcoding validation. Compounds and vehicle are added in a checkerboard pattern to 48 wells, and cells are added and incubated prior to being barcoded using dye gradients of *N*-hydroxysuccinimide functional Pacific Orange (PO) and Pacific Blue (PB). Cells are stained with Ax700, fixed, permeabilized, and pooled prior to immunoassay with antibodies tagged with nonoverlapping fluorescent dyes. Cells are analyzed by flow cytometry and gated selecting (i) intact, single cells, (ii) PO to reveal columns, and (iii) PB to reveal rows and generate populations for each well. PO* and PB* denote dye intensity after correcting for cell morphology using a protocol from ref. ^67^. A data set using Kasumi cells for an etoposide checkerboard assayed with γH2AX binding, which is a marker for DNA damage-associated activity is shown here. **b** Graphical representation of well coordinates generated from pooled cells by gating on forward scatter and γH2AX and then debarcoding as above to reveal the checkerboard pattern. **c** Biaxial plots of individual wells representing a condition, as in Fig. 1a. **d** Percent γH2AX positive for each well was calculated using a cutoff of 10^3.2^ to determine statistical effect size (*Z*′). A similar demonstration with etoposide vs. cCasp3 is shown in Supplementary Fig. 1

To maximize available fluorescence channels for multiparameter flow cytometry, FCB was adapted to barcode 48 wells with two fluorescent NHS ester-activated dye gradients of NHS-Pacific Orange and NHS-Pacific Blue. After two-dimensional barcoding, wells were pooled into a single tube and stained with fluorescently tagged antibodies. To test the robustness of the FCB assay, Kasumi-1 cells were incubated in 48 wells in a checkerboard fashion with vehicle dimethylsulfoxide (DMSO) or one of two benchmark natural products: the podophyllotoxin derivative etoposide^38^, a potent topoisomerase inhibitor and inducer of double-strand DNA breaks, or the bisindole alkaloid staurosporine^39^, a classical inducer of apoptosis. After treatment, cells were stained with a permeability/viability indicator, Alexafluor 700 (Ax700)^40^, barcoded, combined into a single tube, and then stained with fluorescently labeled antibodies specific to either cleaved caspase-3 (cCasp3), a protein activated in apoptosis^41^, or γH2AX, a histone phosphorylated during genomic damage^42,43^. A representative workflow and data for etoposide are shown in Fig. 2. Analysis of single-cell events revealed two populations for each readout, and biaxial plots of Pacific Orange vs. Pacific Blue yielded 48 distinct populations (Fig. 2a). Recovery of the well coordinates and determination of antibody binding in debarcoded populations was accomplished using Cytobank, a cloud-based cytometric analysis platform, to confirm compound-specific effects (Supplementary Fig. 1a, b). In the case of etoposide, gating for γH2AX and then debarcoding illustrated the bifurcated response within the checkerboard (Fig. 2b), and biaxially gated percent changes reflect bioassay results (Fig. 2c). Comparable results were obtained for staurosporine (Supplementary Fig. 1c), and these results were used to calculate the standard deviation of each assay plate, which conformed to levels in standard practice in high-throughput screening analysis (*Z* factor>0.77) (Fig. 2b and Supplementary Fig. 1c). As an additional evaluation of barcoding, cCasp3 and γH2AX expression induced by staurosporine and etoposide, respectively, was demonstrated to be dose dependent within assay conditions by separate cytometric barcoding of concentration response curves and quantitating antibody binding (Supplementary Fig. 1d).

The integrated analysis of an high-performance liquid chromatography (HPLC)/MS-generated chromatographic array in conjunction with FCB and cellular response data (MAM) was validated using a chemically defined mixture of bioactive compounds. A mixture of six structurally and mechanistically diverse cytotoxic small molecules was chromatographically arrayed and assayed against a human myeloid leukemia-derived cell line (KG1) using the MAM platform. EIC chromatograms for the six compounds can be readily compared to bioactivity chromatograms and demonstrated specific and mechanistically expected responses to multiplexed immunoassays (Supplementary Fig. 2a). For instance, the EIC peak for the known apoptosis-inducing secondary metabolite staurosporine (*m/z = *467.5), was the highest correlating peak in the well 25 bioactivity bin for cCasp3. Similarly, the largest response for γH2AX occurred in well 20, matching the retention time of the potent topoisomerase inhibitor etoposide (*m/z* = 606.5). Of note, in this experiment of modest complexity, a single cytometric flow run generates an aggregate of 240 individual raw immunoassays, which may be further combined into additional function assays that can be compared to arrayed compound elution profiles. Importantly, MAM successfully identified and differentiated compounds in a mixture based on their elution profile and differential response to a multiplexed antibody panel.

### MAM finds bioactive metabolites within complex extracts

The identification of bioactive molecules within complex cellular (e.g., microbial) metabolomes using MAM requires that barcoding and bioassay cytometric measurements be stable to potential interferences present under typical secondary metabolite-producing conditions, such as soluble extractable cellular metabolites, cell wall components, and spent growth medium species. The robustness of MAM was therefore tested by fractionating and analyzing a concentrated methanolic microbial extract generated from a *Streptomyces* strain grown in complex media and spiked with etoposide and staurosporine prior to chromatography. Prior to spiking, the extract possessed no measurable bioactivity. After fractionation, wells were evaporated, and KG1 cells were added to the plate and incubated for 16 h. Subsequent to fixation and permeabilization, cells were barcoded, pooled, and assayed using antibodies against γH2AX and cCasp3. Bioactivity chromatograms for these markers were generated from the debarcoded data set and formatted for correlation analysis. Cells were effectively assigned as distinct populations to the 48 wells according to the dye-gradient selection, demonstrating no cytometric interference with FCB from extract components. Moreover, as shown in Supplementary Fig. 2b, plots of median fluorescence intensity for cCasp3, and γH2AX expression per debarcoded population generated bioactivity chromatograms for correlation analysis. Importantly, although the EIC abundance of staurosporine and etoposide was below the threshold of the average intensity of the TIC (Supplementary Fig. 2c), both were the highest Pearson-correlating components in the bioactive fractions^44^. Despite the presence of high-abundance products of cellular metabolism and media components, no additional potent cCasp3 or γH2AX-modulating activities were observed in the test metabolome.

### Finding metabolites with anticancer activity in human tissue

In addition to quantitating intracellular events and cell status immunomarkers, single-cell characterization via cytometry facilitates the differentiation of cell types within heterogeneous mixtures based on cell size, shape, complexity (via differential light scattering), and the detection of cell-type-selective surface markers^45,46^. This enables characterization of the ways in which the components of metabolomic arrays affect molecular phenotypic changes in mixtures of cells, including primary cell preparations that more closely approximate a native cancerous microenvironment than pure immortalized cell lines. Acute myeloid leukemia patient bone marrow samples were selected as an advantageous system for MAM due to their beneficial cytometric properties and clinical significance. AML remains a deadly adult cancer, and treatments have not greatly improved the 5-year overall survival rate, which is 21.3% overall and remains under 5% for patients who are 65 and older^47^. Bone marrow biopsies that are routinely obtained from patients being treated for AML contain a complex mixture of multiple cancer and normal cell types. These tissues are fully “suspended”, require minimal processing (e.g., disaggregation) for cytometric analysis, and contain a mixture of cell types representative of in vivo therapeutic contexts. Cytometric characterization of AML via immunophenotyping is widespread in the diagnosis and management of this disease, providing a strong basis for biomarker selection and analysis.

To test the ability of MAM to assess the effects of a bioactive metabolomic array against a heterogeneous cell mixture, microbial metabolomic arrays were incubated with cell preparations derived from AML biopsy samples from two separate patients. The patient samples used in this experiment represent two common underlying genetic mutational profiles occurring in AML. Patient 001 was a 23-year-old female with a gene translocation (MLL-MLLT3) correlated to intermediate prognosis but without other tested common molecular mutations. Patient 015 was a 68-year-old male with the *FMS*-like tyrosine kinase 3 internal tandem duplication (*FLT3*-ITD) strongly associated with poor prognosis^48^, but with otherwise normal cytogenetics. Subsequent to aspiration from bone marrow, red blood cells and platelets were removed from the patient samples via density gradient separation, resulting in bone marrow mononuclear cells (BMMCs) containing a mixture of heterogeneous AML blasts and nonmalignant myeloid and lymphoid cells and their progenitors^49^. These heterogeneous mixtures served as the response organism system for multiplexed cellular and biochemical analysis. For the microbial metabolomic array source organism, we selected an actinomycete strain designated *Streptomyces specus* that we had isolated from Blue Springs cave in Sparta, Tennessee. *S. specus* was of particular relevance, as it had been observed via depreplication analysis of HPLC/MS data to produce a family of anthracycline natural products related to the clinically employed AML drug daunorubicin, including baumycins, unusual natural acetal functionalized congeners^11^, and related anthracycline functional metabolites with apparent masses not previously reported.

After incubation of the two patient-derived samples with the metabolomic array, cell mixtures were barcoded using the two-color gradient as previously described and pooled. The cellular effects of the array were then analyzed via a six-marker panel: Ax700 (viability), cCasp3 (apoptosis), γH2AX (DNA damage), p-S6 (protein synthesis, growth, and mTOR-mediated metabolism)^50–52^, p-Histone H3 (M phase/proliferation)^53,54^, and CD45 (leukocyte cell surface marker)^55^. After initially gating for intact single cells, BMMC were gated by CD45 expression and side scatter (SSC) to distinguish lymphocytes, myeloid, and leukemia blast cells. The cells in each sample were debarcoded, and markers were quantitated, resulting in 48 debarcoded well populations for blasts, myeloid, and lymphocyte cell types with six readouts for each. Thus, the MAM platform generated bioactivity chromatograms for each cell status marker for each cell type, representing at least 864 unique raw bioassays in a single flow cytometric run, typically acquired in a few minutes. Combined marker analysis via biaxial gating yielded additional phenotypic assays. For instance, combining viability via ±Ax700 and biomarker expression increased the effective number of distinct phenotypic assays per extract up to 36 assays per well, or 1728 per array.

**Fig. 3:**
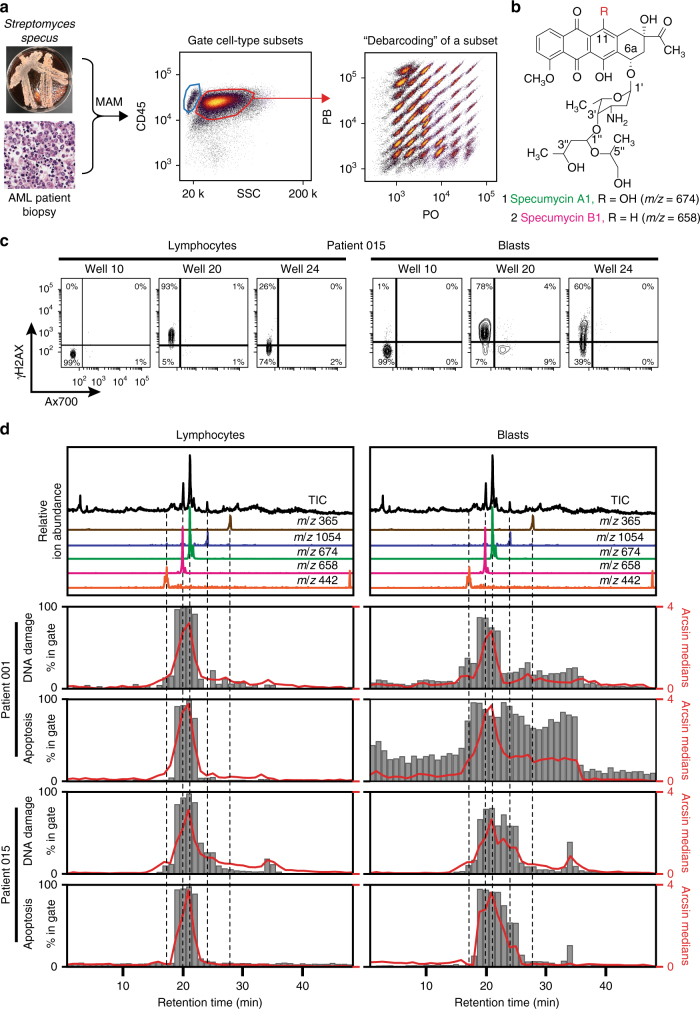
A structurally novel acetal-functional anthracycline selectively targets leukemic blast cells and not nonmalignant lymphocytes within a human bone marrow biopsy. **a** Chromatographic arraying of *Streptomyces specus* was performed using split flow HPLC/UV/MS with polarity-switching mass scanning resulting in an array of highly characterized fractions from a crude extract of the baumycin producer *S. specus*. Primary cell preparations were prepared from AML patient biopsy. The metabolite array was incubated with heterogeneous cell samples, and the cells were then viability stained, fixed, barcoded, and stained with antibodies for biomarker and surface marker expression. Standard gating was performed using biaxial plots of CD45 expression vs. SSC was used to determine cell-type subsets (blue gate: lymphocytes, red gate: blasts) that were each individually analyzed for γH2AX and cCasp3 expression. **b** Structures of specumycin A1 and B1. **c** Biaxial plots of selected wells gated for lymphocytes and leukemia blasts. **d** Total ion current and selected extracted ion currents of metabolites within the metabolome of *S. specus* correlating to bioactive wells from assays against two patient samples. Bar graphs of percent of cells in the upper left quadrant of marker/viability gate (marker positive and viable cells). The solid red line is the arcsinh transformed median of the marker. For patient 015, the average cells collected per well were 3996 blasts (minimum 240) and 764 lymphocytes (minimum 66). For patient 001, the average collected per well was 1655 blasts (minimum 189) and 53 lymphocytes (minimum 17)

In the case of the *S. specus* metabolomic array interacting with AML biopsy samples, and gating for the three major cell types, the strongest bioactivity was observed in the viable (Ax700−) and γH2AX+ and cCasp3+ subsets in both patients (Fig. 3, Supplementary Fig. 3). The sample derived from Patient 015 contained the three readily discernable subpopulations of leukemia blasts, myeloid cells, and lymphocytes, which could be separately debarcoded to yield defined cell-type response profiles in each well (Supplementary Fig. 3a). Patient 001’s sample was comprised of predominantly leukemia blasts and lymphocytes. Individual biaxial plots (Supplementary Figs. 4–5) were used to generate well bioactivity profiles based on set positivity thresholds. A subset of these bioactivity chromatograms is shown in Fig. 3d, and indicates the presence of bioactive molecules in the *S. specus* extract, which can be preliminarily identified via comparison of EIC to bioactivity profiles. Bioactivity profiles represent averages of thousands of single-cell measurements and are highly reproducible between biological replicates (Supplementary Fig. 3b). Two observations result from this data set. First, apparent cell-type selectivity was demonstrated for several features in the metabolomic array. For instance, a 2.5- and 47-fold increase in selective blast-targeting bioactivity vs. leukocytes was observed eluting in wells 17 and 24, respectively. Second, patient-specific activities were evident in bioactivity profiles. Examination of HPLC/MS and bioactivity profiles of well 17, containing the most abundant *m/z* = 442, revealed a 30-fold increase in apoptosis and fivefold increase in DNA damage in patient 001 vs. patient 015. A similar trend was observed in later eluting wells containing anthracycline chromophores. Notably, patient 015 possessed the *FLT3*-ITD phenotype, which is an internal tandem deletion in kinase-encoding gene *FLT3* demonstrated to confer resistance to anthracyclines^48^. Therefore, these observations are consistent with substantially enhanced resistance to anthracyclines in patient 015.

To validate bioactive features putatively identified using MAM, most abundant correlating mass features were isolated. The most potent bioactivity peak was observed in well 21 and correlated to the most abundant eluting anthracycline (*m/z* = 674), termed specumycin A1. Specumycin A1 was isolated in scale-up fermentations and its structure was determined by multidimensional nuclear magnetic resonance experiments (Supplementary Table 2). The planar structure of specumycin A1 is identical to the structures of baumycin A1/2, which contain an unusual acetal appending the 3′-*O*-methyl on the daunosamine sugar^56^. The next most abundant feature, and the primary feature in well 20, was specumycin B1 (Supplementary Table 3), a previously unreported 11-deoxy congener. Specumycin B1 was observed to be as active to A1 under assay conditions but threefold less abundant, suggesting a potentially more potent congener. Comprehensive isolation of low-abundance bioactive species is beyond the scope of this study. However, cell-type and patient-specific responses identified by MAM, such as bioactive metabolites demonstrating enhanced activity against a *FLT3*-ITD AML sample and selective activity for leukemia cells (e.g., well 24, *m/z* = 1054, Fig. 3), demonstrate the potential of this platform for performing preliminary analysis and prioritization of activity differences within a natural product family for common AML subclasses.

**Fig. 4:**
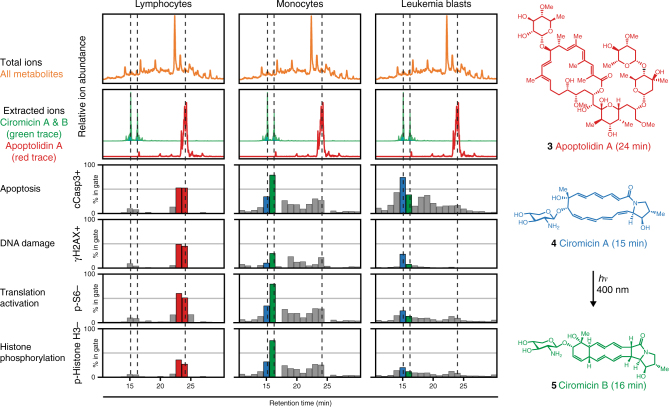
An optochemical cell selectivity switching natural product in *Nocardiopsis* revealed by primary cell MAM. The top metabolome row (orange) shows total ion current and extracted ions for ciromicins A and B (*m/z* = 515, 15, and 16 min) and apoptolidin (*m/z* = 1129.5, 23.4 min) with their elution times shown in dotted lines. The next four rows show selected bioactivity chromatograms from a single flow experiment, which were generated by adding an aspirated preparation of bone marrow mononuclear cells from an AML patient, barcoding, immunostaining, and debarcoding. The immunostaining panel contained CD45 (leukocyte-common antigen), cCasp3 (cleaved caspase), γH2AX (DNA damage), p-Histone H3 (cell cycle marker upregulated during mitosis), and p-S6 (marker for active translation). The average collected per well was 5400 blasts (minimum 209), 750 monocytes (minimum 19), and 291 lymphocytes (minimum 20). Histograms for each marker for highlighted wells are shown in Supplementary Fig. 6

The cell-targeting potential of secondary metabolites within metabolomic arrays in the anthracycline-resistant phenotype sample (015), was further explored employing the soil actinobacterium *Nocardiopsis* sp. FU40 as a source organism. This strain produces a family of bioactive compounds called apoptolidins (A–H)^57^, which are cytotoxic glycosylated macrolides, and a pair of cytotoxic glycosylated polyene macrolactams, ciromicins A and B^58^. Thus, a metabolome array was generated from *Nocardiopsis* sp. FU40, AML Patient 015-derived anthracycline-resistant cell preparations were incubated with the array, and subjected the samples to MAM analysis. In Fig. 4 a selection of bioactivity profiles generated from this single data set is shown, indicating how the arrayed metabolome is obtained from *Nocardiopsis* sp. FU40 can be mined for bioeffectors that have selective activity against different cell types present in an AML patient. For instance, apoptolidins selectively induced caspase-dependent apoptosis in lymphocytes, whereas ciromicins induced apoptosis most prominently in leukemic cells. Similarly, apoptolidin A induced γH2AX, apoptosis, and decreased p-Histone H3 signaling selectively in lymphocytes, and ciromicins induced more DNA damage in monocytes and blast cells. Taken together, these data demonstrate the identification of differential cell targeting of secondary metabolites against primary cell mixtures in the background of an extracted microbial metabolome. In contrast to the specumycins, ciromicins demonstrate potent selectivity for blasts in comparison to lymphocytes in the anthracycline-resistant phenotype.

### Light-modulated secondary metabolite cell-type targeting

Expansion of the bioactivity chromatogram in the region of ciromicin elution revealed that the isobaric metabolites ciromicin A and ciromicin B were resolved into separate wells with strikingly distinct biological phenotypes. Specifically, ciromicin A displayed maximal apoptosis markers in leukemia blast cells, whereas its photoisomerization product ciromicin B stimulated monocyte apoptosis. We recently reported the discovery of ciromicins in an apoptolidin polyketide synthase knockout strain of *Nocardopsis* sp.* FU40*, and demonstrated that ciromicin B is the product of an unexpected visible light-initiated 12-*π* electron photoisomerization of ciromicin A^58^. The identification of ciromicins here in the wild-type monoculture of *Nocardopsis* sp*. FU40* was surprising because it is produced in low levels in the wild-type strain. Thus, the sensitivity of the MAM platform using primary cells was capable of effectively identifying the bioactivity of this low abundant secondary metabolite family, and the modest resolution of 48-well binning of fractions was sufficient to resolve bioactivities of closely eluting species.

**Fig. 5:**
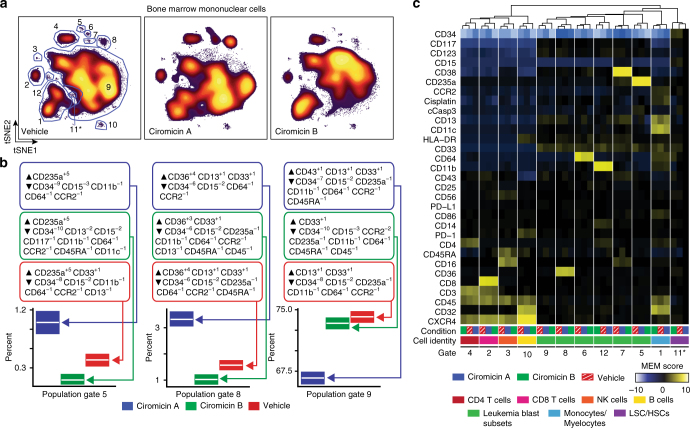
Photochemical isomers ciromicins A and B selectively target different cell subsets within the heterogeneous mixture of patient biopsy cells. Mass cytometry uses DOTA-chelated metals detected by inductively coupled plasma mass spectrometry (ICP-MS) to eliminate spectral overlap, expanding the feature range to 29 antibody-quantified features per cell. **a** viSNE maps of 20,000 individual cells from each condition are organized according to differences in their surface marker profiles for each treatment condition. **b** MEM labels for three blasts subsets and plots of population prevalence with observed prevalence in white and 95% binomial confidence interval represented by a box. **c** Marker enrichment modeling (MEM) was used to characterize major populations within the samples and highlight differences in marker expression relative to a gated population of phenotypic hematopoietic stem cells (gate 11). Heat maps of hierarchical clustered MEM labels reveal subsets specific differences and cellular identification. Heat maps of median marker expression are shown in Supplementary Fig. 7. Dose–response data against PMBCs from a healthy donor and patient sample 015 are shown in Supplementary Fig. 8

The discovery of putative cell-type-specific cellular responses to ciromicin isomers using MAM may be considered as a primary screening “hit”, describing a multidimensional response of a metabolomics fraction with associated correlation coefficient-ranked metabolite features. To validate the unusual photochemically triggered modulation of primary cell selectivity, pure ciromicins A and B were isolated from scaled-up cultures and assayed against the same biopsy sample using an enhanced panel of 29 cell surface markers that classifies all myeloid cell populations when paired with unsupervised machine-learning tools. The viSNE algorithm, which allows robust identification of both nonmalignant and leukemia cell subsets^49^, was applied to data sets collected by mass cytometry after a 48-h treatment of patient-derived BMMC with ciromicin A, ciromicin B, or DMSO. In Fig. 5a viSNE maps showing the overall changes in the cellular landscape of the primary BMMC after this treatment are shown. Proximity in viSNE space corresponds to similarity in cell-type identity, while differences in immunophenotype drive separation of cells (dots) on a viSNE map. To quantify the overall shifts in cellular subsets, gates were drawn on the viSNE map corresponding to prominent populations based on abundance (Fig. 5a). The relative abundance of phenotypically distinct cell subsets present in different treatment conditions and the enriched features of these populations were characterized (Fig. 5a). Per cell marker expression and median values allowed assignment of cellular identity to populations (Supplementary Fig. 7). Changes in the relative abundance of each population demonstrated that photoisomerization polarizes overall cellular immunophenotype within leukemia cells. Based on subset gating within viSNE, ciromicin B reduced the relative abundance of leukemia stem cells and hematopoietic stem cells and smaller blast subsets, while ciromicin A reduced the largest blast subset (subset 9, Fig. 5a). Both isomers had comparatively little impact on lymphoid cells, which were comprised largely of CD4^+^ and CD8^+^ T cells (subsets 2 and 4, Fig. 5a and Supplementary Table 1). Next, marker enrichment modeling (MEM) was used to characterize feature enrichment in comparison to a population of CD34^+^CD38^−^Lin^−^ cells within the leukemia sample. MEM identified changes in populations between ciromicin A and ciromicin B, including greater enrichment for CD13, a marker of myeloid differentiation, and CD43, or sialophorin, which is commonly expressed on more mature myeloid cells such as granulocytes and monocytes, after treatment with ciromicin A within a major leukemia population (subset 9, Fig. 5b). Overall, the pattern of cell-type selectivity expanded the depth of the initial screen, validating MAM’s ability to identify selectively bioactive compounds.

## Discussion

Cancer is challenging to study and to therapeutically manipulate, due in part to the complexity of cell signaling processes affected by pharmacological interactions, and system heterogeneity as seen in the polyclonal nature of cancer cells, the complexity of the supporting stroma, and the infiltrating immune cells^16,20^. MAM as implemented here provides a generalizable system to link metabolomic feature data from one organism or system to functional targets or their causally related networks within another heterogeneous cellular environment. A key feature of the cytometric strategy underpinning MAM is its ability to analyze heterogeneous mixtures of cells, which more closely approximate a native cellular milieu than immortalized cell lines, using multiplexed markers of cell status and type. Specifically, MAM was employed here to study the interkingdom interactions of metabolomes of two secondary metabolite-producing soil bacteria with primary cell preparations from two phenotypically distinct patient-derived AML cell samples. The combination of metabolomics, single-cell biology, and cheminformatics used here identified biologically active secondary metabolites produced at low levels that mediate apoptosis, DNA damage, and cell signaling in a cohort of cells present in AML patient’s bone marrow samples. In this primary cytological screen, differential activities were identified for secondary metabolites present within complex and concentrated microbial extracts.

The cave-derived bacterium *S. specus* is a producer of multiple compounds that share the anthracycline core of daunorubicin, which is used in combination therapy with nucleoside analog cytarabine as the standard of care in the treatment of AML, but differ in decorating glycosides^59^. Specumycins described here are daunorubicin variants similar to baumycins, appended with an unusual acid-labile acetal moiety on the 3′-hydroxyl of daunosamine^60^, and are reported to demonstrate broad cytotoxicity comparable to that of daunorubicin^61^. Despite its clinical significance, daunorubicin is actually a low-abundance biosynthetic intermediate en route to baumycin in most daunorubicin producers, and is typically isolated by acid-catalyzed degradation of baumycin glycosides^60^. However, though being the major product of most daunorubicin biosynthetic pathways, the potential role of the baumycin acetal moiety in cytotoxicity and cell targeting has remained untested prior to this study. Applying MAM to the metabolomic array of *S. specus* revealed activities of a spectrum of specumycin polyketides and related metabolites against divergent primary cell phenotypes. Along with the discovery of the previously unreported and more potent compound specumycin B1, these data suggest previously unnoticed potential for the 3′-acetal functional in AML anthracycline therapy. Validating the observed bioactivity trends, the two most abundant features demonstrated potent activity against leukemia blasts and leukocyte cells, and were isolated and structurally elucidated. Numerous less abundant species displayed remarkable differential cell-type targeting between patients, suggesting an untapped potential for discovery of more selective pharmacological agents within the anthracycline family in biosynthetically competent actinomycete strains.

*Nocardiopsis* sp. FU40 was selected as a subject for the MAM platform as its metabolomic array is complex, both in terms of the sheer number of apoptolidin analogs it produces (denoted A–H), and in its capacity to simultaneously produce polyene macrolactams and aromatic polyketides that were previously reported to possess moderate-to-potent cytotoxicity against cell lines. Applying MAM to test *Nocardiopsis* arrays against AML primary cell preparations successfully deconvoluted apoptolidins from ciromicins and revealed distinct cell-targeting phenotypes. Apoptolidin A and its isobaric analogs present in the extract (isoapoptolidins A and G) correlated to the most potent lymphocyte-targeting activity across all markers. The induction of cCasp3 in lymphocytes is consistent to prior studies of this compound performed in cell lines, which also present evidence in support of mitochondrial FoF1-ATPase targeting within this family^62^. Other apoptolidin congeners are generally chromatographically dispersed from apoptolidin A, but did not display this degree of activity. Notably, the apoptolidins only nominally affected marker expression in leukemia blasts and monocytes, demonstrating how MAM readily identifies first-pass cell-targeting activity in primary tissue samples. The distinctive blast/myeloid cell-type targeting observed for ciromicins A and B was notable, and careful examination of their elution region revealed a remarkable switch of cell specificity between blast and nonblast myeloid lineages for the two compounds.

As a follow-up to using MAM as a primary assay for lead discovery, mass cytometry was used as a secondary validation and deep cell-profiling assay. A 29-marker mass cytometry panel was used to classify the cellular effects of purified ciromicins A and B on subsets in primary cell preparations. Mass cytometry revealed changes in differentiated immunophenotypic subsets and demonstrated that visible light-induced photoisomerization of ciromicin A to B induces wholesale shifts in cell-type targeting and indicating the importance of aglycone structure and geometry to the mechanism of action of this family of macrolactams. For instance, bicyclic Michael acceptor containing ciromicin A exerted its greatest influence on the largest subset of AML cells, whereas tetracyclic potentially less electrophillic ciromicin B targeted stem-like myeloid progenitors, a subset that may be beneficial to address in therapy^63,64^. Mass cytometry also revealed that ciromicins target leukemia blasts in a patient with an anthracycline-resistant leukemia phenotype and, unlike anthracyclines in the previous study, have little negative effect on lymphoid cells. Finally, mass cytometric findings, performed in concert with MAM using patient samples, validated and provided a deeper profiling of bioactive compounds discovered. Overall, the multiplexed single-cell approaches used here represent a paradigm shift in comparison to typical discovery efforts using monoclonal immortalized cell lines or other research models that do not accurately reflect the cell diversity and composition of primary human tumors and leukemic tissues. That ciromicins A and B represent photoswitching natural products with distinct cell subtype-targeting phenotypes provides potential tools for investigating the pharmacology of this family and the effects of targeting subtypes. Notably, molecular photopharmacological switches currently find broad application toward understanding cellular function by leveraging the spatiotemporal control afforded by such compounds^65^.

In summary, a general method is demonstrated for searching preliminary structure–activity relationships in secondary metabolite families in producing organisms, without the need for compound isolation, and provides insight into how bioactive lead compounds affect diseased and normal cell types in major patient phenotypes using clinical samples. Given that there are a limited number of distinct clinical subsets, automated cytometric analysis of untargeted metabolomic inventories against sets of relevant patient phenotypes provides a process for “personalized” natural product discovery. This is a proof-of-principle study of a viable drug discovery platform. In a full-scale implementation, cells derived from multiple patients, including cells derived from healthy individuals, would be necessary to realize the full scope of lead-compound preclinical assessment. While applied here for the case of identifying bioactive secondary metabolites within metabolomes, the MAM platform enables the discovery of cellular responses to molecular inventories, regardless of sources. Given the importance of all chemical communications in mediating life processes within and between organisms, a generalizable method for identifying functional roles for metabolites has significant potential in applications spanning a broad range of applications in cellular chemical biology.

## Methods

### Preparation of microbial crude extracts

*Streptomyces* strains were maintained on ISP2 agar (yeast extract 4 g/L, malt extract 10 g/L, glucose 4 g/L, and agar 20 g/L, pH 7.2). Loops of mycelia were used to inoculate 5-mL seed cultures in ISP2 medium (yeast extract 4 g/L, malt extract 10 g/L, and glucose 4 g/L, pH 7.2) for *Streptomyces* strains, incubating them for 3 days at 30 °C. Seed cultures were then transferred to 250-mL Erlenmeyer flasks containing 25 mL of BA medium (soybean powder 15 g/L, glucose 10 g/L, soluble starch 10 g/L, NaCl 3 g/L, MgSO_4_ 1 g/L, K_2_HPO_4_ 1 g/L, and trace elemental solution 1 mL/L, pH 7.2) and grown for 7 days at 30 °C with shaking. Aqueous fermentation broth was extracted by shaking with Diaion HP20 synthetic absorbent resin (Alfa Aesar) (125 mL of HP20 bead/H_2_O slurry per 500 mL of aqueous broth) for 2 h. Fermentation broth was then centrifuged (3700 × *g*, 30 min) and the supernatant was decanted. Metabolites were eluted from absorbent resin and cells with methanol (250 mL of methanol/125 mL of HP20 bead/H_2_O slurry) by shaking for 1.5 h, followed by centrifugation (3700 × *g*, 30 min) and decanting of the methanol extract. Further extraction was performed with acetone (250 mL of acetone/125 mL of HP20 bead/H_2_O slurry) by shaking for 1.5 h, followed by centrifugation (3700 ×  *g*, 30 min) and decanting of the acetone extract. *Nocardiopsis* strains were cultured and extracted as previously described^57^. Purified ciromicins A and B were isolated from cocultures as previously described^58^. Kasumi-1 and KG-1 cell lines were obtained from ATCC and identified using mass cytometry analysis of 35 myeloid proteins as reported previously^34^.

### Specumycin A1 and B1 isolation

Crude acetone extract was concentrated and fractionated with Sephadex LH-20 resin (GE Healthcare Bio-Sciences) with methanol as the eluent. Fractions were analyzed by analytical HPLC/MS, and fractions containing the compound(s) of interest were pooled and further purified by preparative HPLC (Waters, XBridge C18 Prep, 5 µM) (10 mL/min, 0–1 min: 100% solution A, 5 min: 85% solution A; 15% solution B, 65 min: 15% solution A; 85% solution B, and 70 min: 100% solution B) (Solution A = 95:5, H_2_O:MeCN, 10 mM NH_4_OAc; Solution B: 5:95 H_2_O:MeCN, 10 mM NH_4_OAc). In order to obtain analytical purity, fractions containing the compound of interest (34–35 min) were pooled and purified by flash column chromatography (98:2 CH_2_Cl_2_:MeOH to 95:5 CH_2_Cl_2_:MeOH). The structure of specumycins A1 and B1 was elucidated using a combination of mass spectrometry and two-dimensional nuclear magnetic resonsance spectroscopy data. Mass spectrometry data produced with electrospray ionization and collected in both positive and negative modes provided the molecular weight of specumycins A1 and B1. Correlated nuclear magnetic spectroscopy (COSY) allowed for the assignment of the spin systems present in the aglycone, amino sugar, and acetal moieties of specumycins A1 and B1. Multiplicity-edited heteronuclear single-quantum coherence spectroscopy allowed for assigned 1H shifts to be correlated to their corresponding 13C shifts, as well as for the assignment of shifts as corresponding to methylenes or methines. Full structure elucidation was completed with heteronuclear multiple-bond correlation spectroscopy, which allowed for the assignment of remaining shifts based upon their proximity to assigned shifts.

### Generation of metabolomic arrays

Mass spectrometry was performed by using a TSQ Triple Quantum mass spectrometer equipped with an electrospray ionization source and Surveyor PDA Plus detector. For positive ion mode, the following settings were used: capillary temperature was 270 °C; spray voltage 4.2 kV; spray current 30 mA; capillary voltage 35 V; tube lens 119 V; and skimmer offset 15 V. For negative ion mode, capillary temperature 270 °C; spray voltage 30 kV; spray current 20 mA; capillary voltage 35 V; tube lens 119 V; and skimmer offset 15 V. Fraction plates were prepared by injecting 20 µL of purified compounds in methanol or concentrated extract via a Thermo PAL auto injector onto a phenomenex luna 5 µm C18(2) reverse-phase HPLC column. The sample was fractionated using a gradient of 100% Buffer A (95% H_2_O, 5% acetonitrile) to 100% Buffer B (5% acetonitrile, 95% H_2_O) over 48 min at a flow rate of 1 mL/min and a fixed splitter with a 3:1 ratio with three parts going to the photodiode array detector and fraction collector and one part going to the MS. Fractions were collected in 1-min intervals in a 96 deep well plate. A volume of 150 µL of eluent from each well was transferred to four replica plates and dried in vacuo using a Genevac HT-2 system at 30 °C.

### Fluorescent cell barcoding of cell-seeded metabolomic arrays

Eight serial 1:2.14 dilutions of Pacific Blue were prepared, covering a concentration range from 0.038 to 7.67 μg/mL. Six serial 1:2.5 dilutions of Pacific Orange were prepared, covering a concentration range from 0.22 to 21 μg/mL. Each dilution of Pacific Blue was added to all wells in a single row of a 96-well plate (10 μL/well), so that the dye concentration in each row decreased from the top to the bottom of the plate. Similarly, each dilution of Pacific Orange was added to all wells in a column of the same 96-well plate (10 μL/well), so that the concentration in each column decreased from columns 1 to 6 and from columns 7 to 12. This procedure yielded two sets of 48 barcoded wells per plate. Approximately 200,000 cells (180 µL suspended in phosphate-buffered saline (PBS)) were added to each well and incubated in the dark at room temperature for 30 min. Staining was then quenched by addition of 75 µL of 1% BSA (Sigma) in PBS.

### Antibody staining

Cells were stained with antibodies in 100-μL staining medium for 30 min in the dark, unless otherwise noted. Individual antibodies were added in accordance with the manufacturer’s instructions. Staining was quenched with 1% BSA in PBS, and stained cells were washed with PBS prior to analysis.

### Validation checkerboard

Kasumi-1 cells were incubated with either 20 μM etoposide or 1 μM staurosporine and/or DMSO in a checkerboard pattern overnight. After treatment, cells were stained with Alexa 700, fixed, permeabilized, barcoded, pooled, and then stained with anti-γH2AX-PerCP-Cy5.5 (clone N1-431, BD) or anti-cleaved caspase-3-PE (clone C92-605, BD). Subsequent to staining, samples were run on a five-laser BD Fortessa flow cytometer. Upon gating single-cell events, wells were debarcoded, and the percent of positive cells for each respective marker was determined for each of the 48 populations. *Z* scores were calculated according to the formula *Z* = 1 – 3(*σ*_p_ + *σ*_n_)/|*μ*_p_ – *μ*_n_|.

### Dose–response curves

Serial dilutions of etoposide and staurosporine were prepared from DMSO stocks (10 mM) covering a range from 100 μM to 100 nM. A volume of 1 μL of each dilution point was added to a well. Each compound titration was handled individually on a separate plate. KG1 cells [150,000 in 199 µL of culture medium (RPMI1640 + 20% FBS + 1% penicillin/streptomycin)] were added to each well and mixed by pipetting. After incubation for 16 h, cells were stained with Alexa 700, fixed with 1.6% paraformaldehyde, and permeabilized in methanol for 20 min at –20 °C. Wells were then barcoded as described above, combined, and then stained with antibodies specific for anti-cleaved caspase-3-PE (clone C92-605, BD), or anti-γH2AX-PerCP-Cy5.5 (clone N1-431, BD).

### MAM protocol with six pure compounds

DMSO stocks (10 mM) of etoposide, staurosporine, CL994, PF04708671, PCI34051, mevastatin, and NU7441 were added (1 µL each) to 43 µL of methanol and fractionated as described above. Before addition of cells, compounds in the wells were suspended by addition of 1 µL of DMSO and mixing by Vortex. KG1 cells [150,000 in 199 µL of culture medium (RPMI1640 + 20% fetal bovine serum (FBS) + 1% penicillin/streptomycin)] were added to each well and mixed by pipetting. After incubation for 16 h, cells were stained with Alexa 700, fixed with 1.6% paraformaldehyde, and permeabilized in methanol for 20 min at −20 °C. Wells were then barcoded as described above, combined, and then stained with the following antibodies: anti-cleaved caspase-3-PE (clone C92-605, BD), anti-p-Histone H3-PE-Cy7 (clone HTA28, BioLegend), anti-γH2AX-PerCP-Cy5.5 (clone N1-431, BD), and anti-p-S6-Ax647 (clone D57.22E, CST).

### MAM using crude extract with internal standards

DMSO stocks (10 mM) of etoposide and staurosporine were added (1 µL each) to 48 µL of a crude extract (200 mg/mL in 50% methanol/water) and fractionated as described above. The extract was generated from a *Streptomyces* cave strain grown in BA medium, and extracted with 50% methanol prior to evaporation in vacuo. Before treatment, compounds in the wells were suspended by addition of 1 µL of DMSO and mixing by vortexing. KG1 cells [150,000 in 199 µL of culture medium (RPMI1640 + 20% fetal bovine serum (FBS) + 1% penicillin/streptomycin)] were added to each well and mixed. After incubation for 16 h, cells were stained with Ax700, fixed with 1.6% paraformaldehyde, and permeabilized in methanol for 20 min at −20 °C. Wells were then barcoded as described above, combined, and then stained with anti-cleaved caspase-3-PE (clone C92-605, BD) and anti-γH2AX-PerCP-Cy5.5 (clone N1-431, BD).

### AML patient samples

All specimens were obtained in accordance with the Declaration of Helsinki following protocols approved by the Vanderbilt University Medical Center Institutional Review Board. Details of patients and sample acquisition were previously published^49^. Briefly, consent was obtained via an approved written consent form, and eligibility criteria included ≥18 years of age with suspected AML undergoing clinical evaluation at Vanderbilt. Samples analyzed here were collected from bone marrow prior to any treatment. Once obtained, samples underwent immediate (within <30 min) density gradient separation of mononuclear cells using a BD Vacutainer CPT Cell Preparation Tube with Sodium Heparin (BD Biosciences, Franklin Lakes, NJ). The separated mononuclear cells were then pelleted with low-speed centrifugation (200 × *g*) and aliquoted into multiple cryotubes in an 88% FBS + 12% DMSO solution. Samples were stored at −80 °C for 24–72 h prior to long-term storage in liquid nitrogen. Patient 1,305,001 (001) was found to have the MLL-MLLT3 t(9;11)(p22;q23) translocation in all cells by karyotype analysis and was without other tested common molecular mutations (which included *FLT3*-ITD, NPM1, CEPBA, and c-KIT)^66^. Patient 1305015 (015) had a normal karyotype, but was found to have both a *FLT3*-ITD and an NPM1 mutation.

### MAM of bacterial extracts against primary cell preparations

Primary cell preparations were thawed, and 200,000 cells were added to each well of a fraction plate containing a metabolite array that was generated from a crude extract from *S. specus* or *Nocardiopsis* sp. FU40. After a 16-h incubation, cells were stained for viability, fixed, permeabilized, barcoded, and stained with the following antibodies: anti-Human CD45-Ax488 (clone HI30, BioLegend), anti-cleaved caspase-3-PE (clone C92-605, BD), anti-p-Histone H3-PE-Cy7 (clone HTA28, BioLegend), anti-γH2AX-PerCP-Cy5.5 (clone N1-431, BD), and anti-p-S6-Ax647 (clone D57.22E, CST). SSC and CD45 expression were used to define lymphocyte, monocyte, and blast populations. Each population was then debarcoded, and readouts were determined for 48 wells per cell type.

### Deep profiling of ciromicins A and B against primary cell preparations

Ciromicins were purified by separation on a Water 600 HPLC system with a reverse-phase column using a linear gradient of water/acetonitrile containing 0.1% formic acid. Fractions with UV absorbance indicative of ciromicins were then combined and applied on a size exclusion Sephadex LH-20 column for a gravity elution in methanol. Ciromicin A and B were then separated by a secondary HPLC purification. Approximately 6 million cells (2 million per condition) from a thawed primary AML sample were incubated in culture medium [80% RPMI 1640 (Mediatech, Inc., Manassas, VA) + 20% FBS (Gibco standard FBS, Life Technologies, Grand Island, NY) with 10 μM ciromicin A, ciromicin B, or DMSO] for 48 h. Mass cytometry experiments were performed as previously described^17^. Briefly, after incubation with ciromicin A, B, or vehicle, samples were pelleted by centrifugation at 200 × *g*, resuspended and washed with PBS (HyClone, HyClone Laboratories, Logan, UT), pelleted, and resuspended in PBS. They were then stained with Cell-ID Cisplatin (Fluidigm, South San Francisco, CA) as per the manufacturer’s recommended protocol. The cells were washed and resuspended in staining medium [CSM: PBS + 1% BSA (Fisher Scientific, Fair Lawn, NJ)]. Cells were then stained with a mass cytometry antibody panel of 29 extracellular antibodies designed to characterize AML blasts and most non-AML peripheral blood mononuclear cells consisting of anti-human CD235a-141 (clone HIR2, Fluidigm), anti-human CD117-143 (clone 104D2, Fluidigm), anti-human CD11b-144 (clone ICRF44, Fluidigm), anti-human CD4-145 (clone RPAT4, Fluidigm), anti-human CD64-146 (clone 10.1, Fluidigm), anti-human CD36-147 (clone 5-271, BioLegend), anti-human CD34-148 (clone 581, Fluidigm), anti-human CCR2-149 (clone K036C2, BioLegend), anti-human CD43-150 (clone 84-3C1, Fluidigm), anti-human CD123-151 (clone 6H6, Fluidigm), anti-human CD13-152 (clone WM15, Fluidigm), anti-human CD45RA-153 (clone HI100, Fluidigm), anti-human CD45-154 (clone HI30, Fluidigm), anti-human CD86-156 (clone IT2.2, Fluidigm), anti-human CD33-158 (clone WM53, Fluidigm), anti-human CD11c-159 (clone BU15, Fluidigm), anti-human CD14-160 (clone M5E2, Fluidigm), anti-human CD32-161 (clone FUN-2, BioLegend), anti-human CDHLA-DR-163 (clone L243, BioLegend), anti-human CD15-164 (clone W6D3, Fluidigm), anti-human CD16-165 (clone 3G8, Fluidigm), anti-human CD38-167 (clone HIT2, Fluidigm), anti-human CD8-168 (clone SK1, Fluidigm), anti-human CD25-169 (clone 2A3, Fluidigm), anti-human CD3-170 (clone UCHT1, Fluidigm), anti-human CD184-173 (clone 12G5, Fluidigm), anti-human PD1-174 (clone EH12.2H7, Fluidigm), anti-human PD-L1-175 (clone 29E.2A3, Fluidigm), and anti-human CD56-176 (clone CMSSB, Fluidigm)^50^. A master mix of these antibodies was added to each sample to give a final staining volume of 50 µL and incubated at room temperature for 30 min. Cells were then washed twice, first with CSM and then with PBS and then permeabilized in –20 °C 100% methanol for 20 min. Following permeabilization, cells were washed, stained with 250 nM iridium intercalator (Fluidigm, South San Francisco, CA) for 30 min at 4 °C, washed twice in PBS, and then resuspended in 500 µL of ddH_2_O for CyTOF analysis. Samples were analyzed using a CyTOF 1.0 cytometer (Fluidigm, South San Francisco, CA)^49^.

### Data availability

Kasumi validation data (Fig. 2) and AML patient sample responses to *S. specus* (Fig. 3) and *Nocardiopsis* (Fig. 4) were generated by fluorescence cytometry as described by Irish et al^19,20^. and Krutzik et al^24,26,35^. and are available as FCS files in FlowRepository (https://flowrepository.org/experiments/1476, https://flowrepository.org/experiments/1477, https://flowrepository.org/experiments/1478, and https://flowrepository.org/experiments/1479). AML patient sample responses to ciromicin A and ciromicin B (Fig. 5) were generated by mass cytometry as described by Leelatian et al^67^. and Ferrell et al^34^. and are available as FCS files in FlowRepository (https://flowrepository.org/experiments/1480). MEM enrichment scores (Fig. 5b) were generated as in Diggins et al^36^. using stem-like population 11 as a reference.

## Electronic supplementary material

Supplementary Information
