# Supplementary material for: Discovery of human cell selective effector molecules using single cell multiplexed activity metabolomics
Source: Nat Commun. 2018 Jan 2;9:39. doi: 10.1038/s41467-017-02470-8 (PMC5750220; doi:10.1038/s41467-017-02470-8)

Supplementary Figure 1.

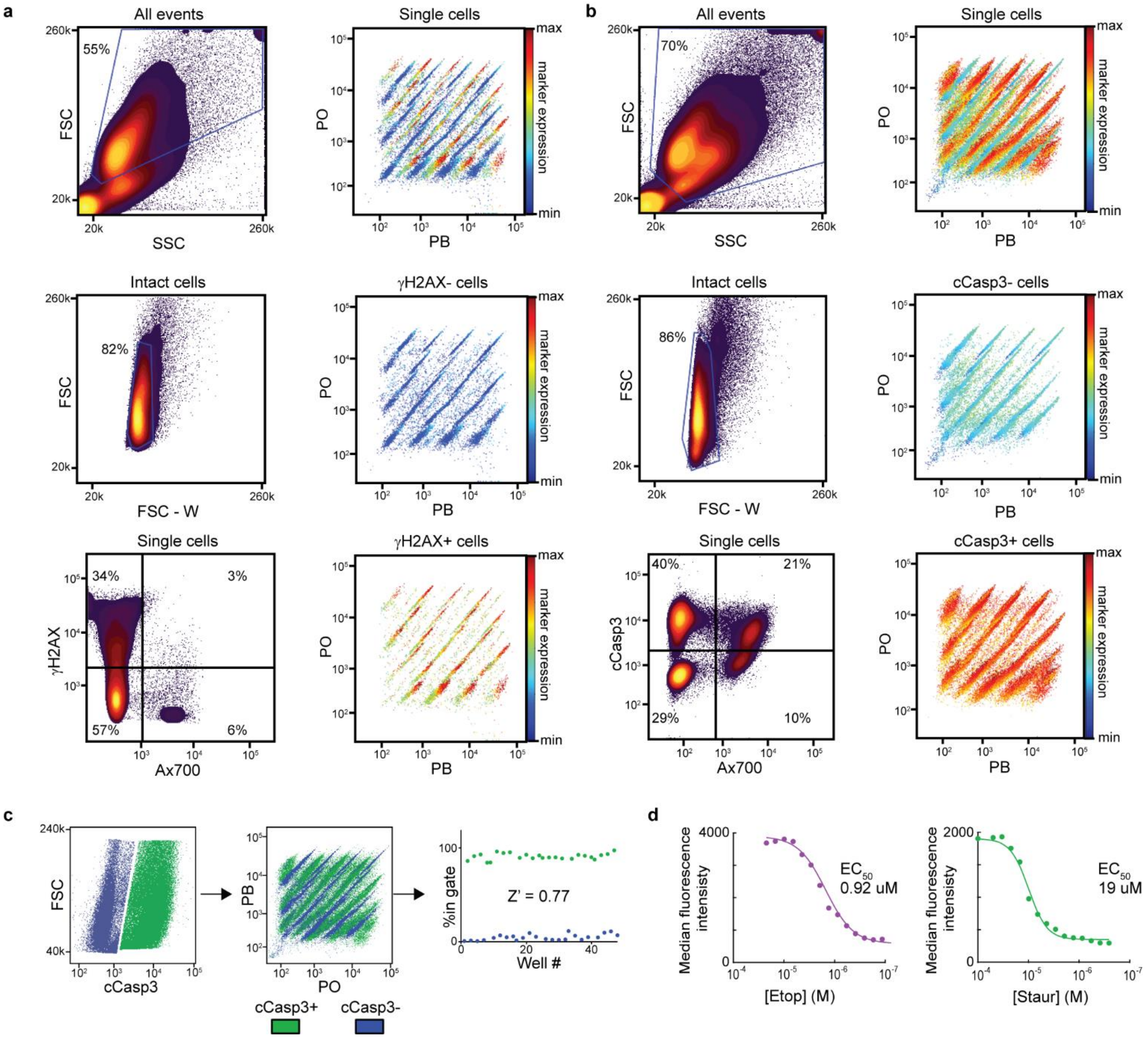

Supplementary Figure 1. Gating strategy for assay validation and demonstration of dose dependent behavior in FCB array assays. a. All collected events from etoposide checkerboard experiment were gated for intact cells (FSC vs SSC), then single cells (FSC vs FSC-W) and finally for maker expression. Biaxial plots of single cells (PO vs PB) colored by γH2AX expression visually reflect the checkboard pattern. Plots of only γH2AX- cells or γH2AX+ cells show populations whose FCB coordinates match assay wells with vehicle or compound respectively. **b**. Same as **(a)** for staurosporine checkerboard experiment with cCasp3 used as the marker readout. **c**. Determination of z score from staurosporine checkerboard. **d**. A titration series of etoposide and staurosporine was prepared on a microtiter plate, incubated with KG1 cells, barcoded and stained. EC_{50} values were calculated using the median fluorescent intensity of cell populations from each well. The average collected cells per well used to calculate z score was 1016 events (minimum 79). The average collected cells per well used to calculate EC_{50} values was 3122 events (minimum 794).

Supplementary Figure 2.

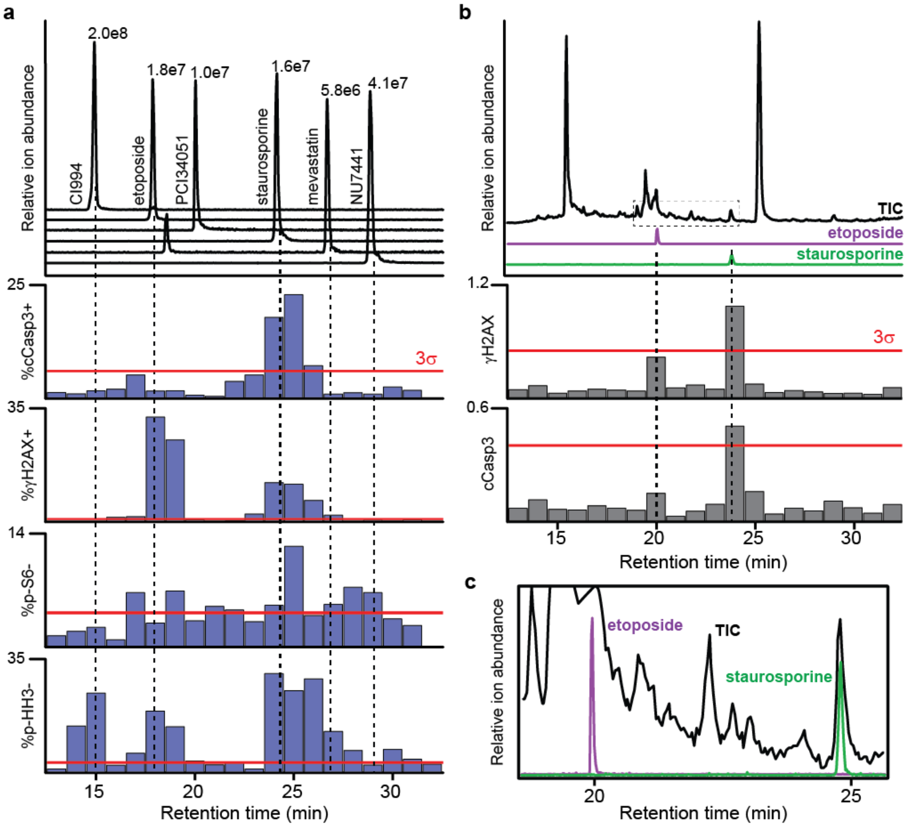

Supplementary Figure 2. **Integration and validation of chromatographic arrays and FCB**. Chromatographic arraying is performed using split flow HPLC/UV/MS with polarity switching mass scanning resulting in an array of highly characterized fractions. **a**. A mixture six bioactive small molecules was arrayed onto a microtiter plate via split flow HPLC/MS fractionation and solvent was evaporated. Subsequently KG1 cells were added to the wells of the plate for incubation with the various toxicants. Cells were stained with Alexa-700 dye to indicate cell viability, fixed, permeabilized, barcoded, pooled and then immuno-stained with antibody-dye conjugates for DNA damage and apoptosis using anti-γH2AX and anti-cCasp3, respectively, and additional conjugates directed against phosphorylated Histone H3 (p-HH3), and phosphorylated ribosomal protein S6K (p-S6). The sample was analyzed via flow cytometry, and reconstructed bioactivity chromatograms were generated by gating on viable and marker positive (γH2AX and cCasp3) or marker negative (p-Histone H3 and p-S6) cells. Selective ion traces are aligned with bioactivity chromatograms. **b**. crude extract spiked with etoposide and staurosporine prior to fractionation. Bioactivity chromatograms were constructed using the arcsinh transformed median of all cells per well. **c**. expansion of TIC, and EIC for etoposide and staurosporine. Red line denotes threshold for signal greater than 3 standard deviations of the readout from 4 blank control wells. The average collected cells per well in **a** was 656 events (minimum 89) and in **b** was 1277 (minimum 102).

Supplementary Figure 3

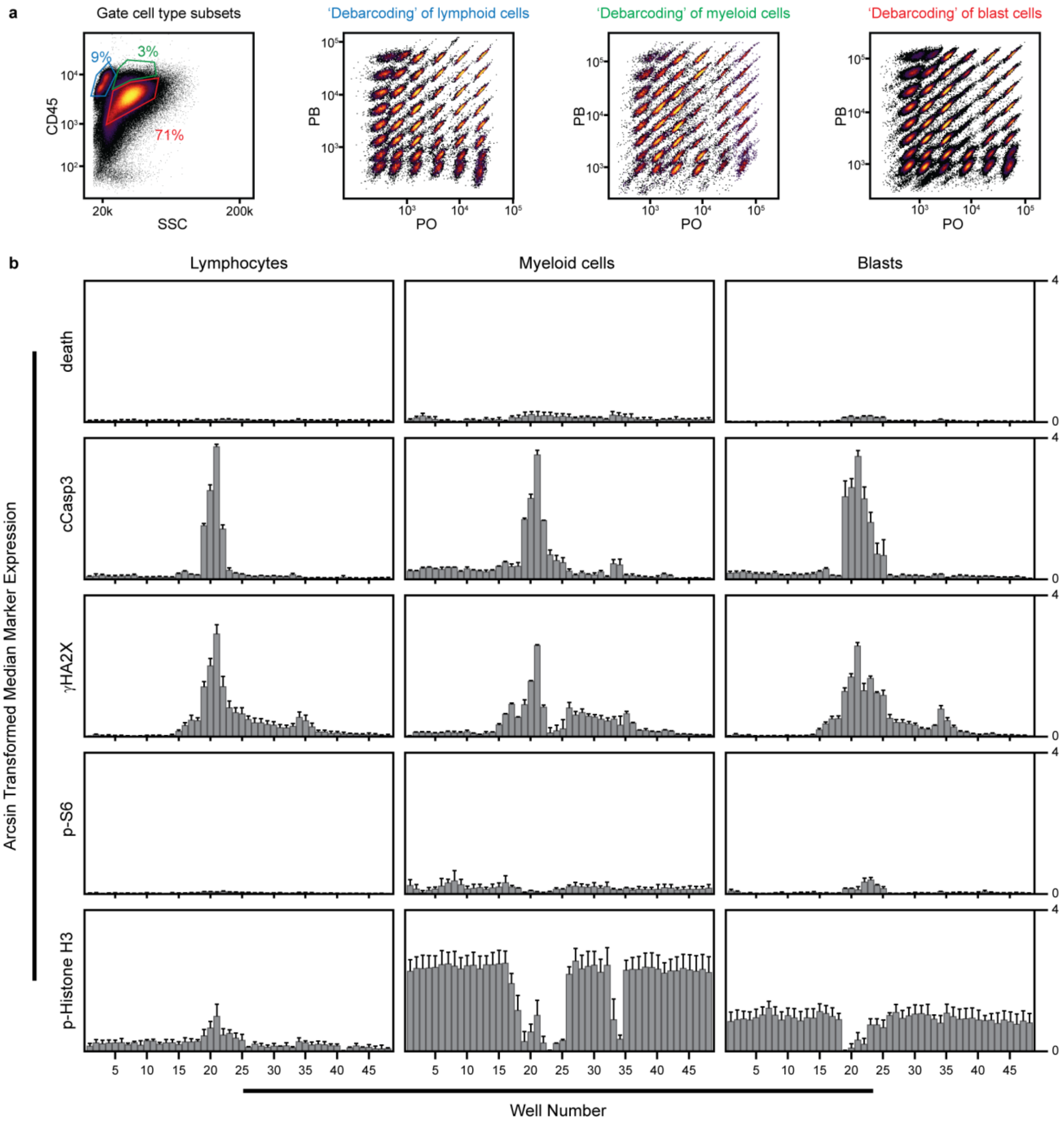

Supplementary Figure 3. Comparison of replicates of MAM with primary patient samples. Samples from patient 015 were incubated with two replicate plates of the fractionated *S. specus* extract. Bar graphs show the average of the arcsin transformed medians for each marker for the two experiments. Error bars are standard deviations. For each of the 48 lymphocyte populations from each well, an average of 1360 events were recorded in each replicate experiment. For myeloid populations the average collected cells per well was 595 and for blast populations was 5755.

Supplementary Figure 4.

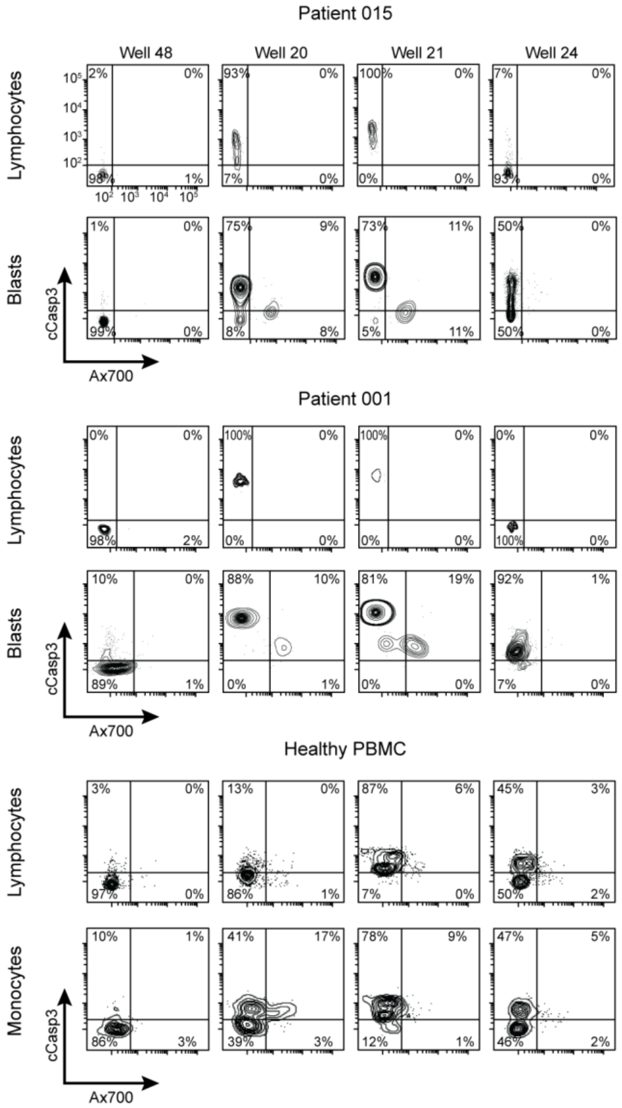

Supplementary Figure 4. Biaxial plots of cCasp3 vs Ax700 from fraction wells containing specumycins. The predominant analogs of the specumycins elute from 20 to 21 minutes with well 21 containing the highest amount of specumycin A. Also shown are the biaxial plots from well 48 which contained only elution buffer and well 24 containing the *m/z* 1054.

Supplementary Figure 5.

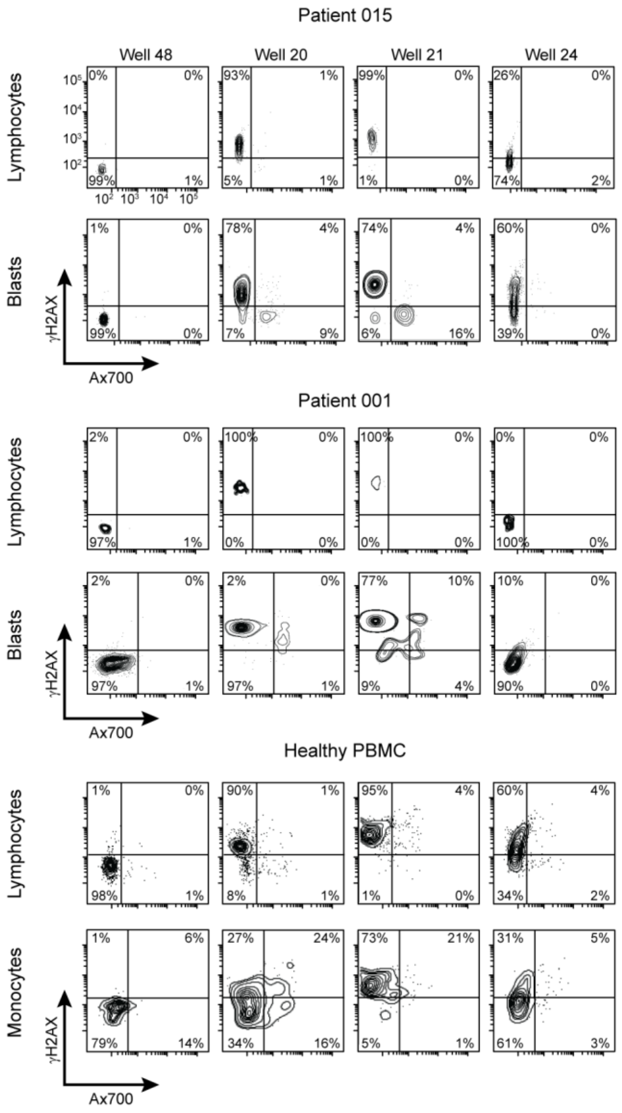

Supplementary Figure 5. Biaxial plots of γH2AX vs Ax700 from fraction wells containing specumycins. Same as Supplementary Figure 4. except corresponding plots for γH2AX are shown instead.

Supplementary Figure 6.

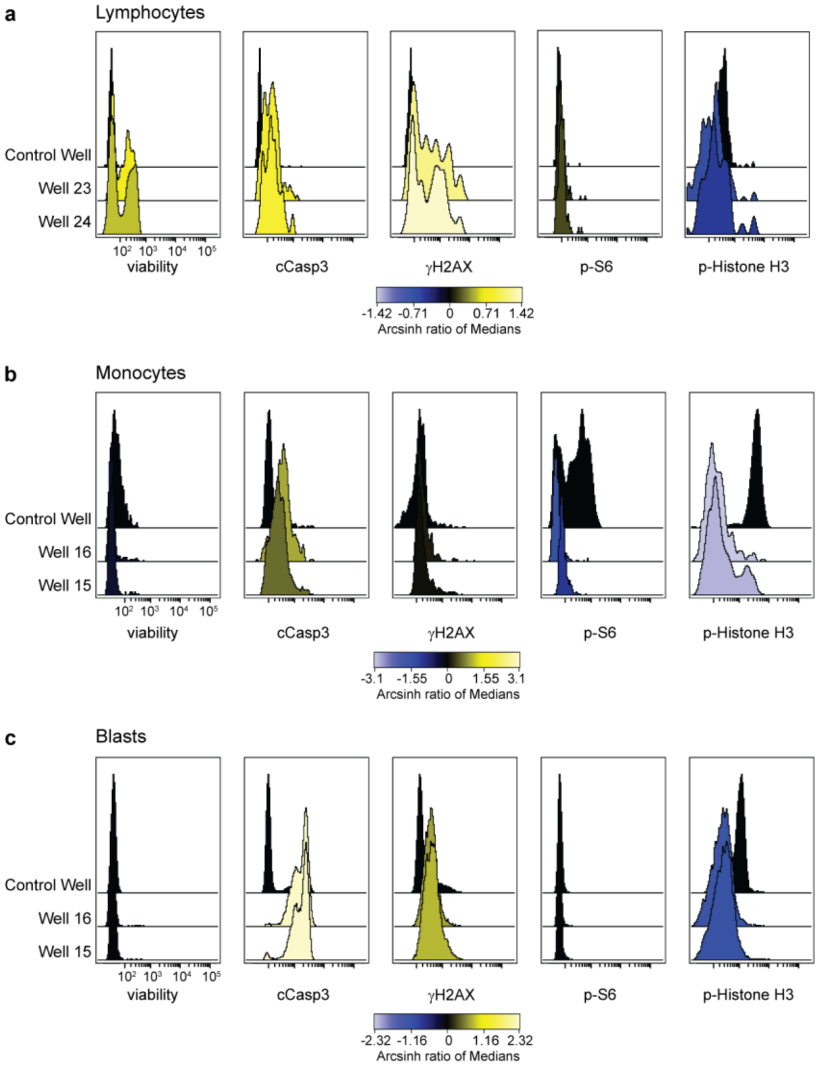

Supplementary Figure 6. Histogram plots of each marker in control wells and highlighted wells in Figure 4. a Marker distribution in wells 23 and 24 which had the maximal response in lymphocytes and contained the apoptolidins. **b** and **c** Marker distribution in wells 15 and 16 which contained the ciromicins which induced the largest response in monocytes and blasts.

Supplementary Figure 7.

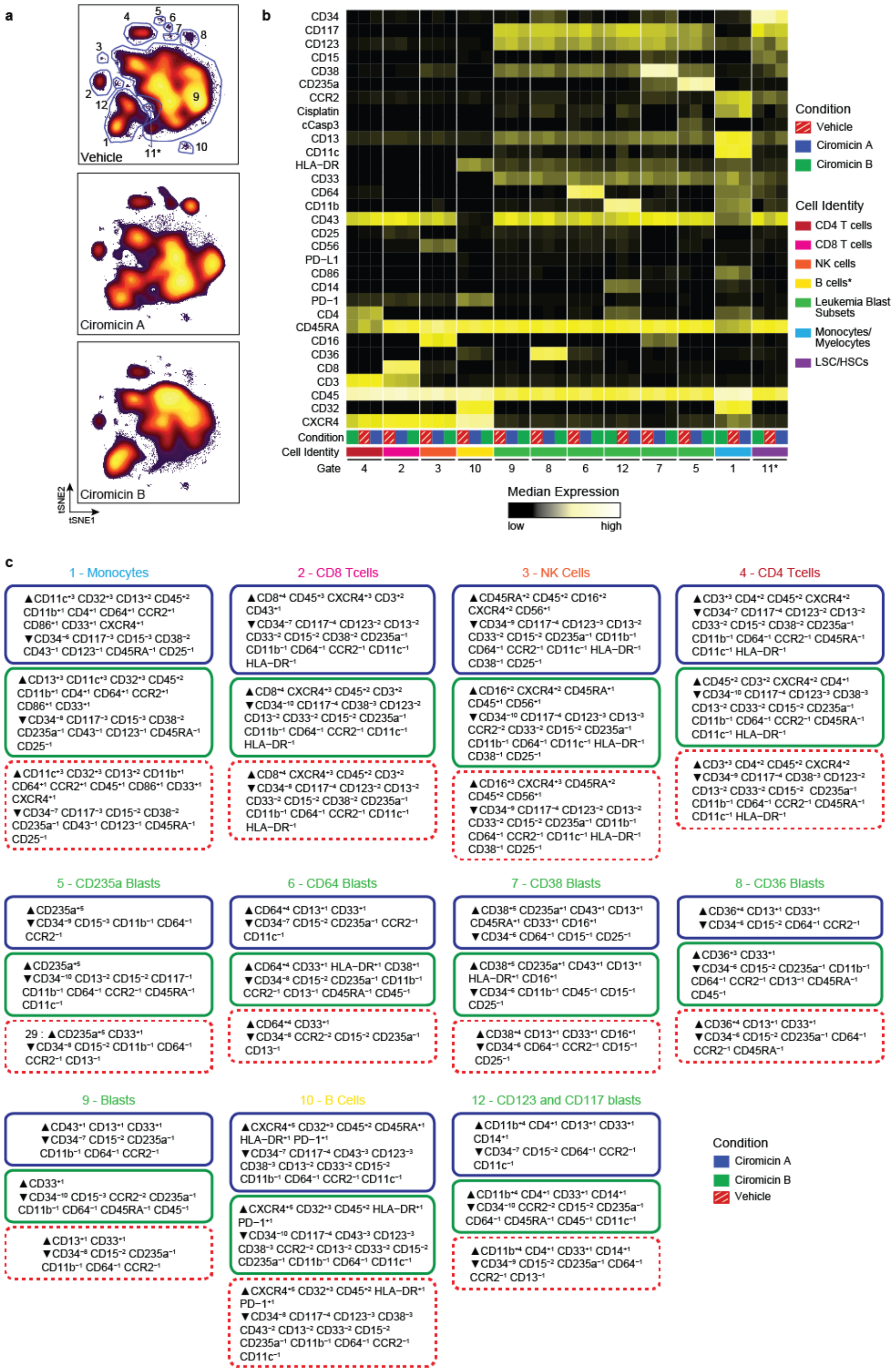

Supplementary Figure 7. Median marker expression of gated viSNE populations. a 12 major populations were identified after viSNE analysis and gated. **b** Median marker expression for each gated population after treatment with vehicle, ciromicin A or B. **c** MEM text labels for gated populations with population 11 (LSC/HSCs) used as reference.

Supplementary Figure 8.

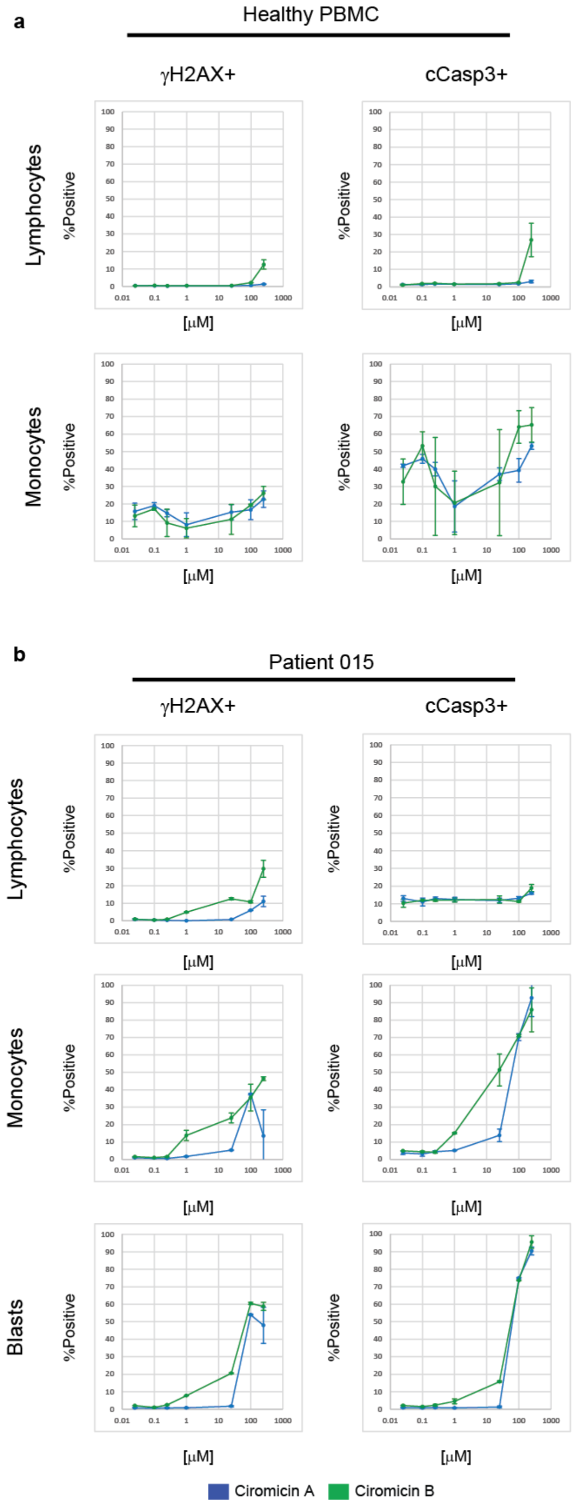

Supplementary Figure 8. 24 hour titration of ciromicins assayed against **a** PBMCs from a healthy donor and **b** a AML patient sample. Points represent the average of two replicate experiments.

Supplementary Table 1. Changes in Population (Percent of Cells) in Response to Ciromicins

Gate	Vehicle	Ciromicin A	Ciromicin B
1	13.6	16.0	17.1
2	2.8	2.9	2.4
3	0.3	0.4	0.3
4	4.3	3.6	4.1
5	0.4	1.0	0.1
6	0.5	0.8	0.2
7	0.3	1.2	0.2
8	3.6	3.3	1.0
9	73.5	66.0	72.9
10	0.9	1.0	0.9
11	0.4	1.3	0.1
12	0.5	1.0	0.4

Supplementary Table 2. NMR Shift Assignments for specumycin A1

Position	^{1}H NMR δ_H *(J in Hz)*	^{13}C NMR δ_C	^{1}H - ^{1}H COSY NMR δ_H	^{1}H - ^{13}C HMBC NMR δ_C
1	7.89 d (5.9)	120.1	7.71	187.2, 136.1, 121.6, 118.9
2	7.71 t (7.8)	136.0	7.89, 7.32	161.8, 136.1
3	7.32 d (7.8)	118.9	7.71	187.2, 161.8, 120.1
7	5.17	69.8	2.27, 2.08	135.3, 77.6
8	2.27 d (14.6),<br>2.08 dd (14.8, 4.2)	35.4	5.17, 3.16	77.6, 69.8, 33.8
10	3.16 d(18.6),<br>2.89 d (18.6)	33.8	2.27	212.6, 156.3, 135.1, 77.6, 35.4
14	2.40 s	25.4		212.6, 77.6
1'	5.5	100.5	3.82, 1.93	67.8, 46.8
2'	1.93-1.88 m	32.7	5.50, 3.35	46.8
3'	3.35	46.8	3.82, 1.93	74.2
4'	3.82	74.2	5.50, 4.15, 3.35, 1.93	100.5, 46.8, 32.7, 17.6
5'	4.15 q (6.4)	67.8	3.82, 1.29	100.5, 74.2, 46.8, 17.6
6'	1.29 d (6.7)	17.6	4.15	74.2, 67.8
1''	4.84	101.2	1.86	
2''	1.86-1.81 m	42.4	4.84, 4.23	101.2, 64.4
3''	4.23 m	64.4	1.86, 1.20	
4''	1.20 d (6.2)	24.2	4.23	64.4, 42.4
5''	3.76	73.0	3.54, 3.50, 1.13	73.0, 16.6
6''	3.54 dd (11.8, 2.1)<br>3.50 dd (11.8, 7.1)	66.5	3.76	
7''	1.13 d (6.3)	16.6	3.76	73.0, 66.5
4		161.8	7.71, 7.32	
5				
6				
9		77.6	5.17, 3.16, 2.89, 2.40	
11		156.3	3.16, 2.89	
12		187.2	7.89, 7.32	
13		212.6	3.16, 2.89, 2.40	
4a		121.6	7.89, 7.32	
5a				
6a		135.3	5.17	
10a		135.1	3.18, 2.89	
11a				
12a		136.1	7.89, 7.71	
4-OMe	4.01	57.2	7.32	161.8

Supplementary Table 3. NMR Shift Assignments for specumycin B1

Position	^{1}H NMR δ_H *(J in Hz)*	^{13}C NMR δ_C	^{1}H - ^{1}H COSY NMR δ_H	^{1}H - ^{13}C HMBC NMR δ_C
1	7.88 d (7.6)	120.5	7.71, 7.32	182.8, 120.9, 118.8
2	7.71 t 8.0)	136.1	7.88, 7.32	161.3
3	7.32 d (8.4)	118.8	7.88, 7.71	188.7, 161.3, 120.5
7	5.14	69.8	2.25, 2.09	130.0, 77.8
8	2.25 d (14.6), 2.09 dd (14.8, 4.1)	36.1	5.14, 3.00	130.0, 77.8, 69.8, 39.7
10	3.15 d (17.4), 3.00 d (17.4)	39.7	7.41, 2.25	212.6, 143.1, 130.0, 120.0, 77.8, 36.1
14	2.37 s	25.4		212.6, 77.8
1'	5.48	99.8	3.85, 2.06, 1.99	69.8, 67.1, 46.8
2'	2.06, 1.99	30.2	5.48, 3.56	
3'	3.56	46.8	3.85, 2.06, 1.99	
4'	3.85	72.6	4.17, 3.56	100.7, 46.8, 30.2
5'	4.17 q (5.9)	67.1	1.27	99.8, 72.6, 17.5
6'	1.27 d (6.6)	17.5	4.17	72.6, 67.1, 30.2
1''	4.78	100.7	1.87, 1.80	
2''	1.87, 1.80	41.4	4.78, 4.25	100.7, 64.0
3''	4.25	64	1.87, 1.80, 1.18	
4''	1.18 d (6.1)	24.4	4.25	64.0, 41.4
5''	3.7	72.6	3.62, 3.51, 1.14	100.7, 66.1
6''	3.62 dd (18.7, 8.0), 3.51 d (10.8)	66.1	3.7	72.6
7''	1.14 d (6.0)	16.2	3.7	72.6, 66.1
4		161.3	7.71, 7.32, 4.00	
5		188.7	7.32	
6				
9		77.8	5.14, 3.15, 3.00, 2.37, 2.25, 2.09	
11	7.41 s	120	3.15, 3.00	188.7, 182.8, 130.0, 39.7
12		182.8	7.88	
13		212.6	3.15, 3.00, 2.37	
4a				
5a				
6a		130	5.14, 3.15, 3.00, 2.25, 2.09	
10a		143.1	3.15, 3.00	
12a		120.9	7.88	
4-OMe	4.00 s	57.1	7.32	161.3, 118.8

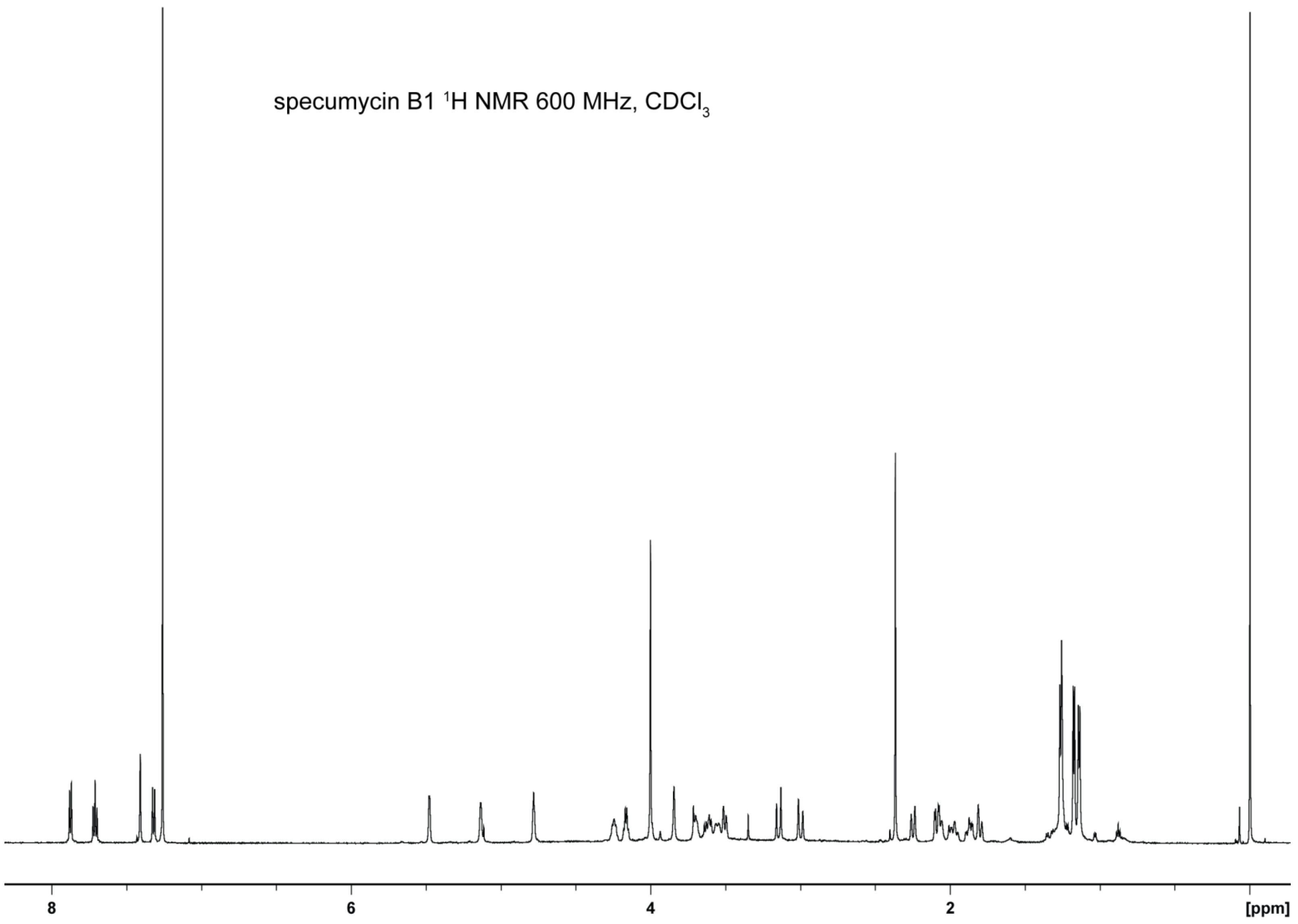
specumycin B1 ^{1}H NMR 600 MHz, $CDCl_3$
8
6
4
2
[ppm]

specumycin B1 COSY NMR 600 MHz, $CDCl_3$

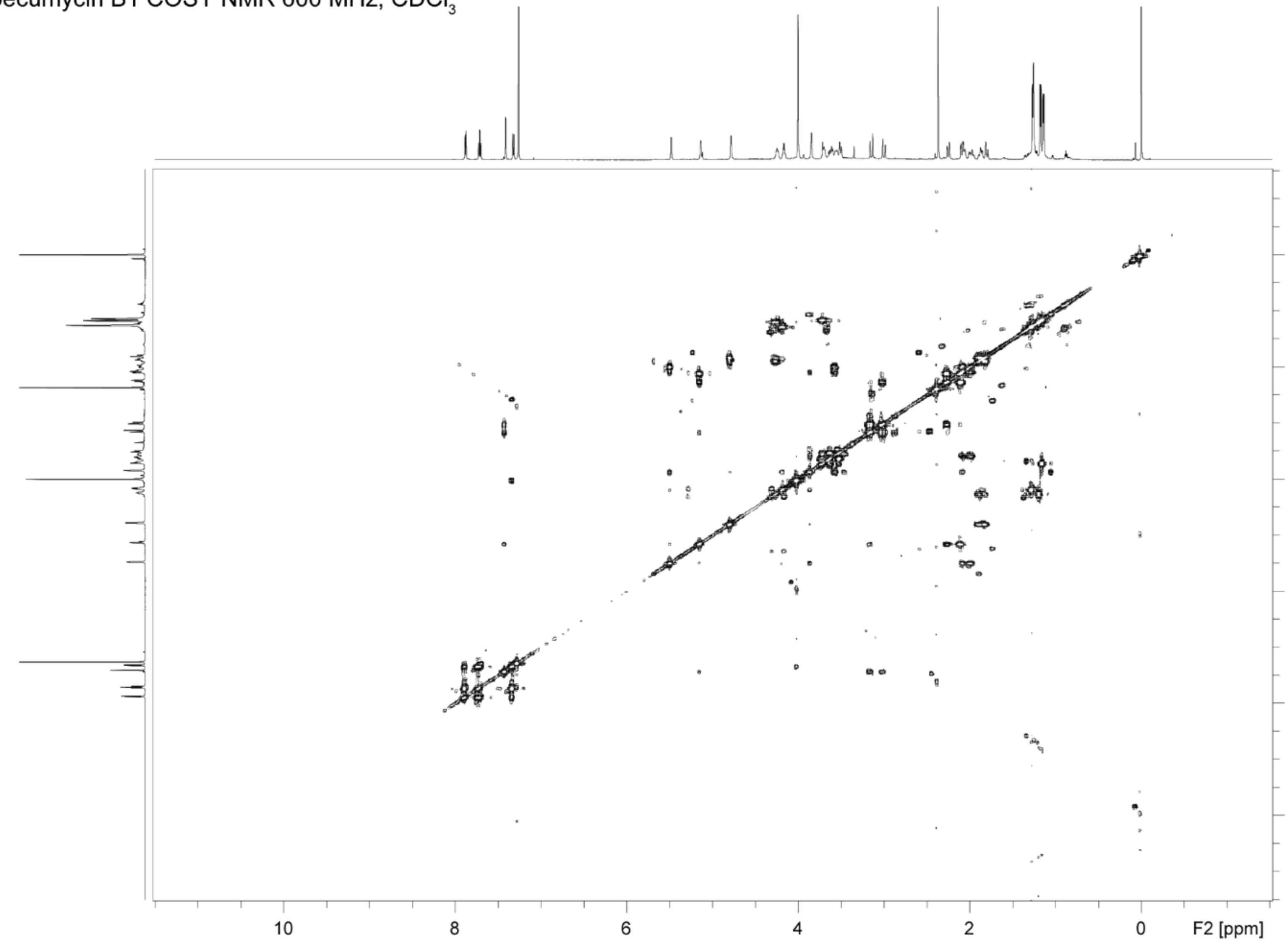

specumycin B1 HSQC NMR 600 MHz, $CDCl_3$

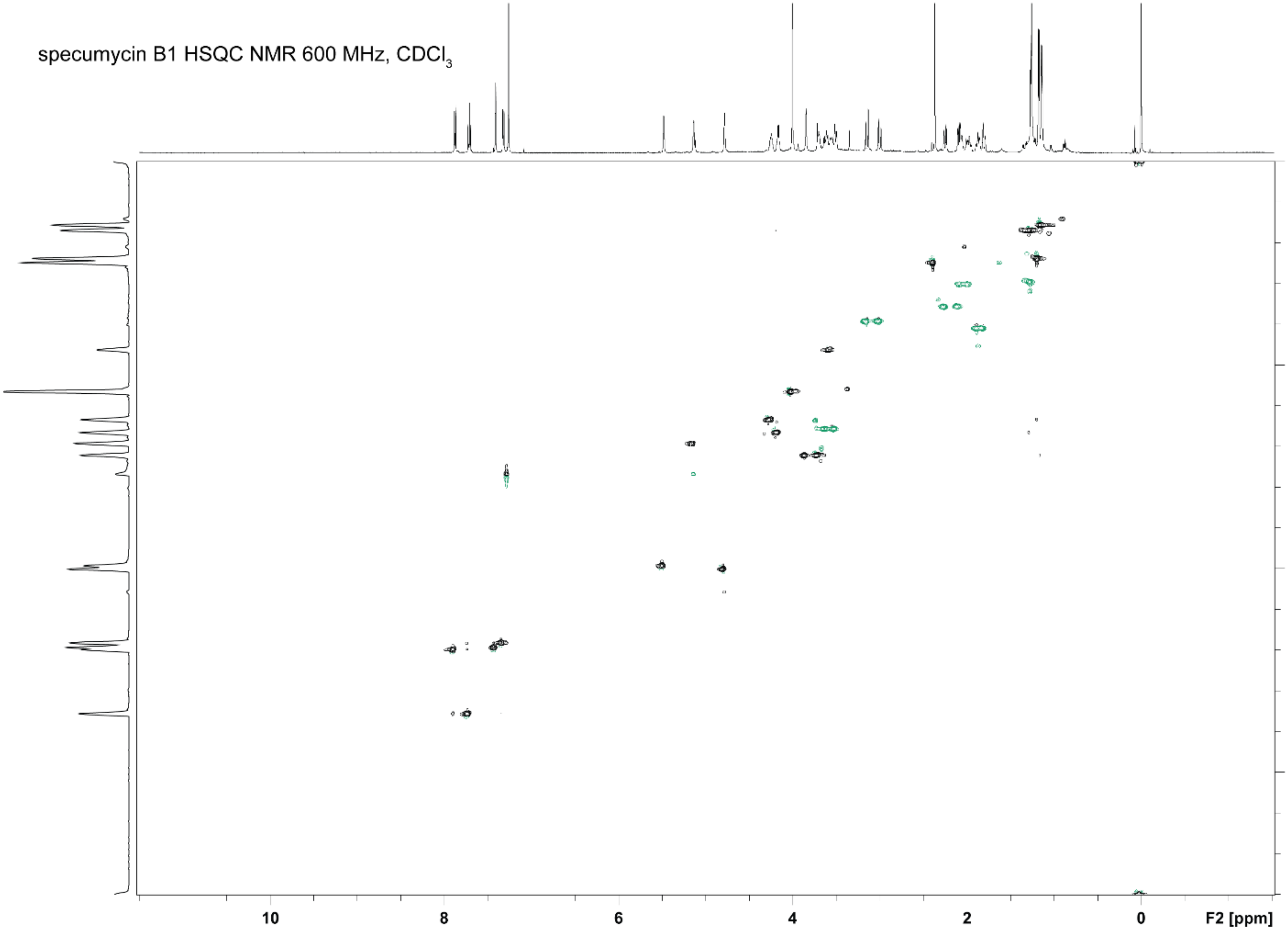

specumycin B1 HMBC NMR 600 MHz, $CDCl_3$

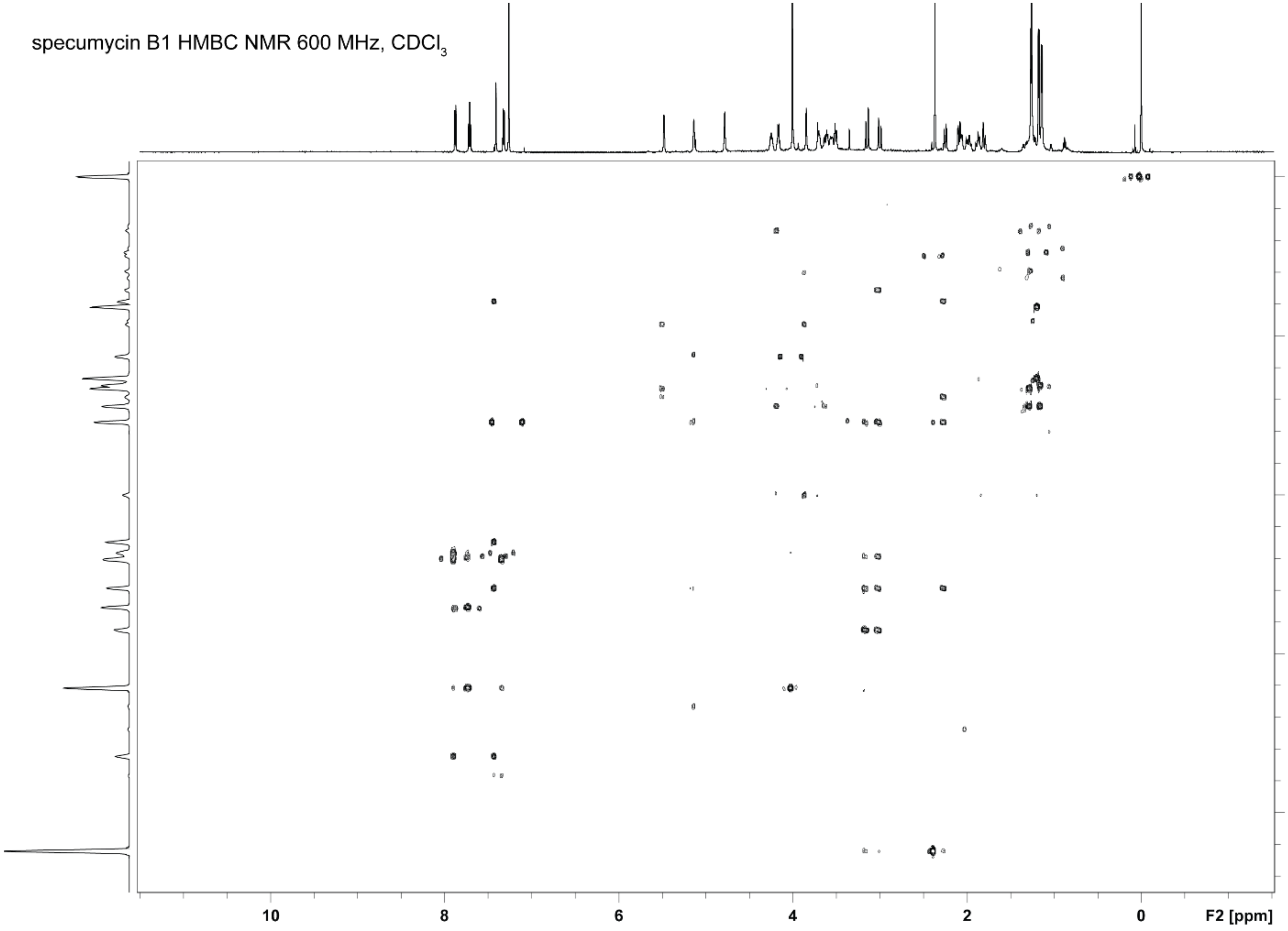

Supplement: Supplementary file 1 — Supplementary Information [file 41467_2017_2470_MOESM1_ESM.pdf]
